# Prediction of Phase Equilibria of Polycyclic Aromatic Hydrocarbons in Organic Solvents with the SAFT‑γ Mie Equation of State

**DOI:** 10.1021/acs.iecr.6c02192

**Published:** 2026-09-21

**Authors:** Thomas Bernet, Shubhani Paliwal, Benjamin I. Tan, Andrew J. Haslam, Claire S. Adjiman, George Jackson, Amparo Galindo

**Affiliations:** Department of Chemical Engineering, Sargent Centre for Process Systems Engineering, 4615 Imperial College London, South Kensington Campus, London SW7 2AZ, United Kingdom

## Abstract

Polycyclic aromatic hydrocarbons (PAHs) constitute an essential family of compounds for the petroleum and material industries, but also a major cause of environmental pollution and health issues. Finding approaches to mitigate their impact requires a better understanding of their thermophysical properties, which presents a challenge because of their structural complexity and diversity. We use the SAFT-γ Mie group-contribution approach to calculate the properties of PAHs in various organic solvent families, including alkanes, alcohols, ketones, esters, and aromatic compounds. Following recent work [ TanB. I., . Energy Fuels 2025, 39, 2435–2470 ], where an aromatic carbon (aC) group was developed, we characterize 33 new group interactions of SAFT-γ Mie using data of PAHs and styrene derivatives. Excellent agreement is found between the calculations and experimental data, including for the solid–liquid equilibria of 193 binary mixtures. The new group interactions can also be used to model pharmaceuticals and agrochemicals.

## Graphical abstract

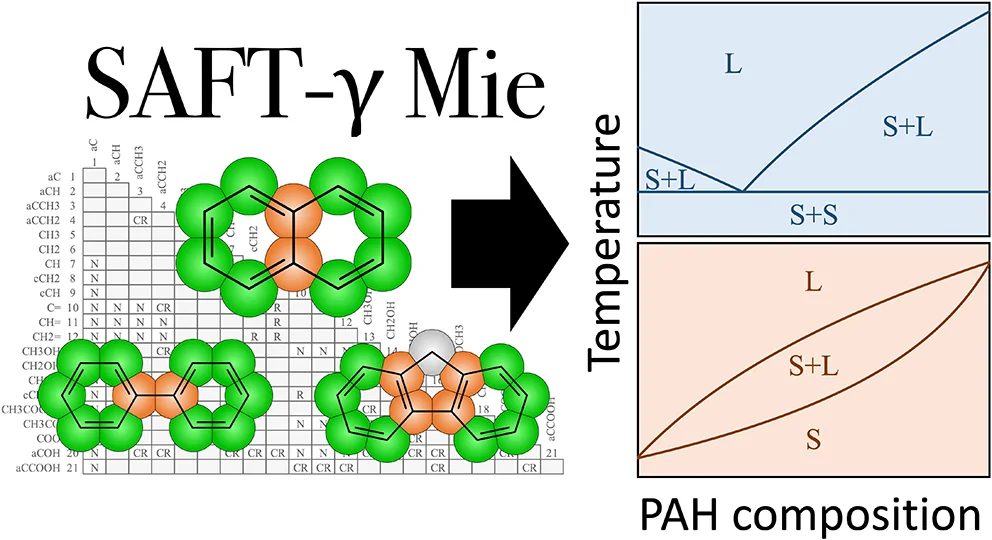

## 1 Introduction

Delocalization of electrons in unsaturated compounds significantly affects their physical and thermodynamic properties, including phase behavior. Unsaturated bonds within rings introduce additional stability due to conjugation effects. Aromatic compounds, which contain fully conjugated π-electron systems, are particularly noted for their enhanced thermodynamic stability^1^ leading to higher melting points and distinct chemical properties, such as hydrophobicity.^2^ Benzene and its derivatives serve as prototypical examples of aromatic hydrocarbons, demonstrating unique electronic structures^3,4^ that contribute to their widespread industrial significance.^5–7^ Partial saturation of the aromatic rings leads to compounds that exhibit intermediate properties between fully saturated and fully aromatic structures; they retain some degree of electronic delocalization but also introduce new stereochemical properties, such as higher conformational flexibility.

Furthermore, the presence of cis/trans isomerism in partially saturated rings can significantly impact melting points, solubilities (of a solid in a liquid), and phase behavior, as steric hindrance and electronic effects influence molecular packing and intermolecular forces. Additionally, the polarity of substituents on aromatic and partially aromatic compounds and functional groups such as hydroxyl, carboxyl, or halogen atoms modulate dipole moments and intermolecular interactions, thereby influencing the phase behavior of the mixtures containing these compounds.

Polycyclic aromatic hydrocarbons (PAHs) represent a class of compounds with multiple aromatic rings (or multiple phenyl groups) fused together (Figure 1). The interplay of π–π stacking interactions, molecular size, and van der Waals forces can lead to complex phase behaviors that are key to their practical applications. Understanding the phase behavior of PAHs in organic solvents is essential in numerous applications, including petrochemical processing,^8,9^ environmental science,^10–12^ and materials engineering.^13,14^ In particular, mixtures that contain PAHs in the solid phase can exhibit various types of behavior, such as eutectic systems^15^ or complete miscibility in the solid phase.^16^ Accurately predicting their phase equilibria is crucial for optimizing separation processes, designing new solvents, and assessing environmental impact. Beyond PAHs, the functional groups found in PAH + organic solvent mixtures are found in many other industrially relevant mixtures such as mixtures of organic solvents with pharmaceuticals, agrochemicals, and other fine chemicals. Thus, characterizing the corresponding group–group interactions is an essential step in modeling these important mixtures.

**Figure 1:**
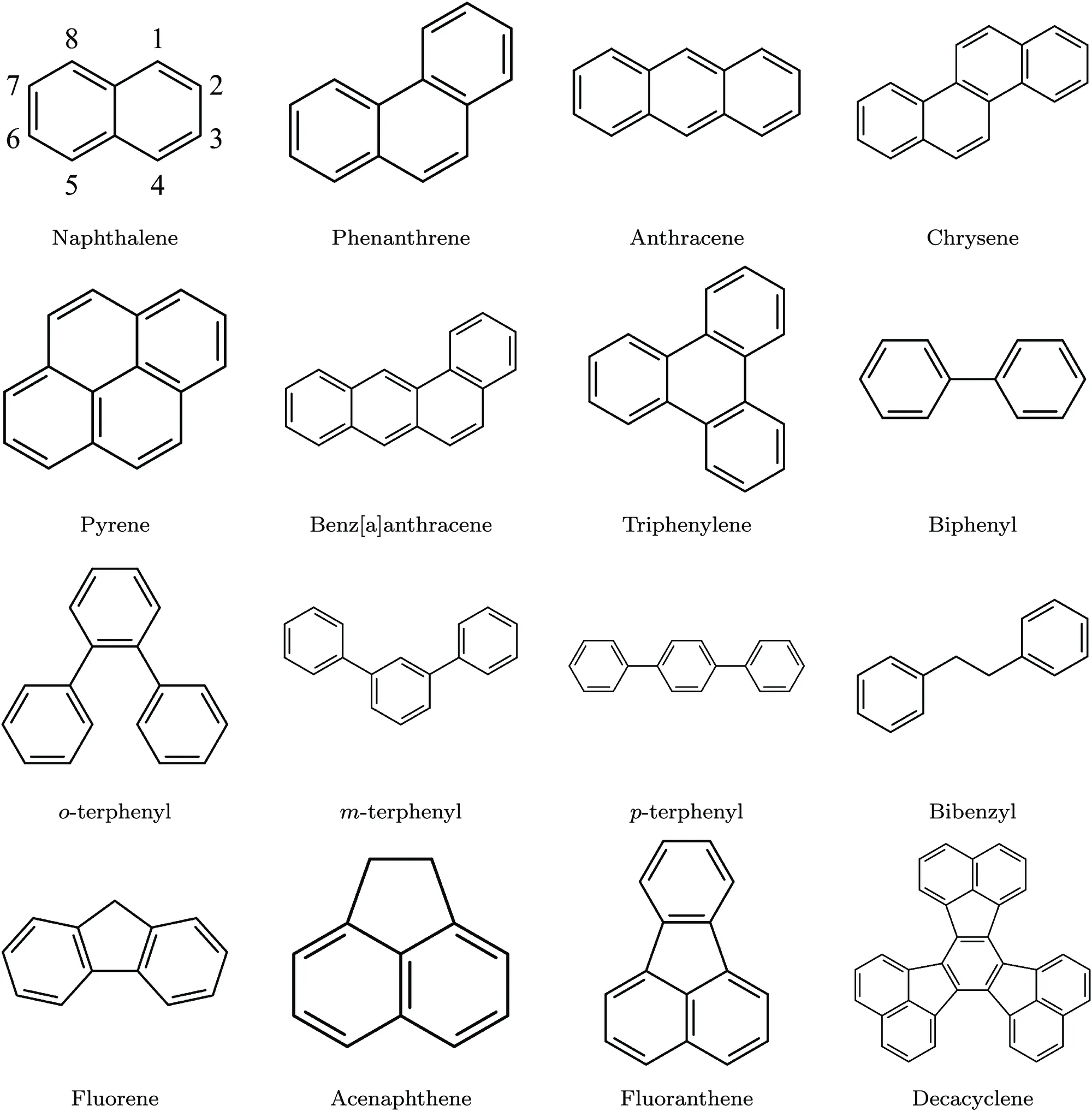
Molecular structures of the polycyclic aromatic hydrocarbons (PAHs) considered in our work. The nomenclature for the structure of naphthalene indicate the positions of the methyl groups defining the alkylnaphthalenes considered (i.e., 1-methylnaphthalene, 2-methylnaphthalene, 2,3-dimethylnaphthalene, 2,6-dimethylnaphthalene, and 2,7-dimethylnaphthalene).

In the current work, we study the phase behavior of PAHs in organic solvents using a variant of the statistical associating fluid theory (SAFT)^17,18^ referred to as SAFT-γ Mie,^19–22^ by extending the recent work of Tan et al.^23^ The SAFT-γ Mie^19–22^ equation of state is a group-contribution method developed to extend the predictive capability of the SAFT-VR approach.^24–26^ Instead of specifying interactions between entire molecules, interactions between functional groups or combinations of functional groups are defined in the SAFT-γ Mie model, which significantly improves its applicability to predict properties of new complex fluids, including compounds that have never been synthesized before or for which no experimental data are available. In addition to the original SAFT-γ Mie version, we note that another group-contribution treatment for branched compounds, known as the s-SAFT-γ Mie version,^27–32^ has been developed. Group-contribution methods like SAFT-γ Mie are particularly useful in molecular and process design to minimize experimental effort and material consumption while enabling the study of systems across a wide range of thermodynamic conditions.

For pure fluids, the most common thermodynamic properties studied with SAFT-γ Mie are the vapor pressure, saturation density, and vaporization enthalpy, and in some instances derivative properties such as heat capacity and isothermal compressibility. For mixtures, the types of phase behavior studied include vapor–liquid equilibrium (VLE), liquid–liquid equilibrium (LLE), solid–liquid equilibrium (SLE), or combinations thereof; liquid density and excess molar enthalpy can also be considered for mixtures. These thermodynamic properties have been studied with SAFT-γ Mie for several families of complex fluids, including pharmaceutical compounds,^33–35^ fragrance compounds,^36–38^ amino acids and peptides,^39^ polymers,^40,41^ cholesterol,^42^ and Li-ion batteries.^43^ In the recent work by Tan et al.,^23^ SAFT-γ Mie has been used to study the thermophysical properties of systems considered in the petroleum industry, including the vapor pressure and saturation density of pure PAHs, the VLE of PAHs + monocyclic aromatic hydrocarbons and PAHs + linear alkanes, and the VLE and LLE of asphaltenes + monocyclic aromatic hydrocarbons and asphaltenes + linear alkanes. An alternative SAFT-γ Mie group representation and parametrization has also been independently developed by Velásquez Sarmiento and Paricaud^44^ for PAHs and functionalized polycyclic aromatic compounds. For consistency with our previous work, we consider the publication of Tan et al.^23^ as a basis for our current work.

A SAFT-γ force field is also available to study PAHs with molecular simulation.^45^ In addition, it is important to mention that VLE calculations for PAH systems have been considered with other versions of SAFT.^46–50^ The SLE of PAHs in organic solvents have been studied with alternative theoretical methods, such as UNIFAC-based^51–61^ and COSMO-based^60–64^ approaches. Alternative approaches exist, such as the cubic-plus-association (CPA) equation of state, which has been applied to various hydrocarbons and PAHs.^65–68^

Here, we extend the group parametrization of the SAFT-γ Mie approach to model the thermodynamic properties and phase behavior of pure PAHs that have not been considered yet with SAFT-γ Mie, as well as binary mixtures involving PAHs. To the best of our knowledge, we present the most extensive comparison of PAH + solvent experimental data and calculations to date, in terms of both the number of mixtures and the number of data points. The mixtures considered encompass a range of organic solvent families, including alkanes, primary and secondary alcohols, ketones, esters, and functionalized aromatic compounds. Several monocyclic aromatic hydrocarbons and unsaturated aliphatic compounds are also considered to characterize and validate new group-interaction parameters that are relevant to the fluids of interest. The exhaustive list of compounds and the nomenclature used to refer to the main solvent families can be found in Figure 2.

**Figure 2:**
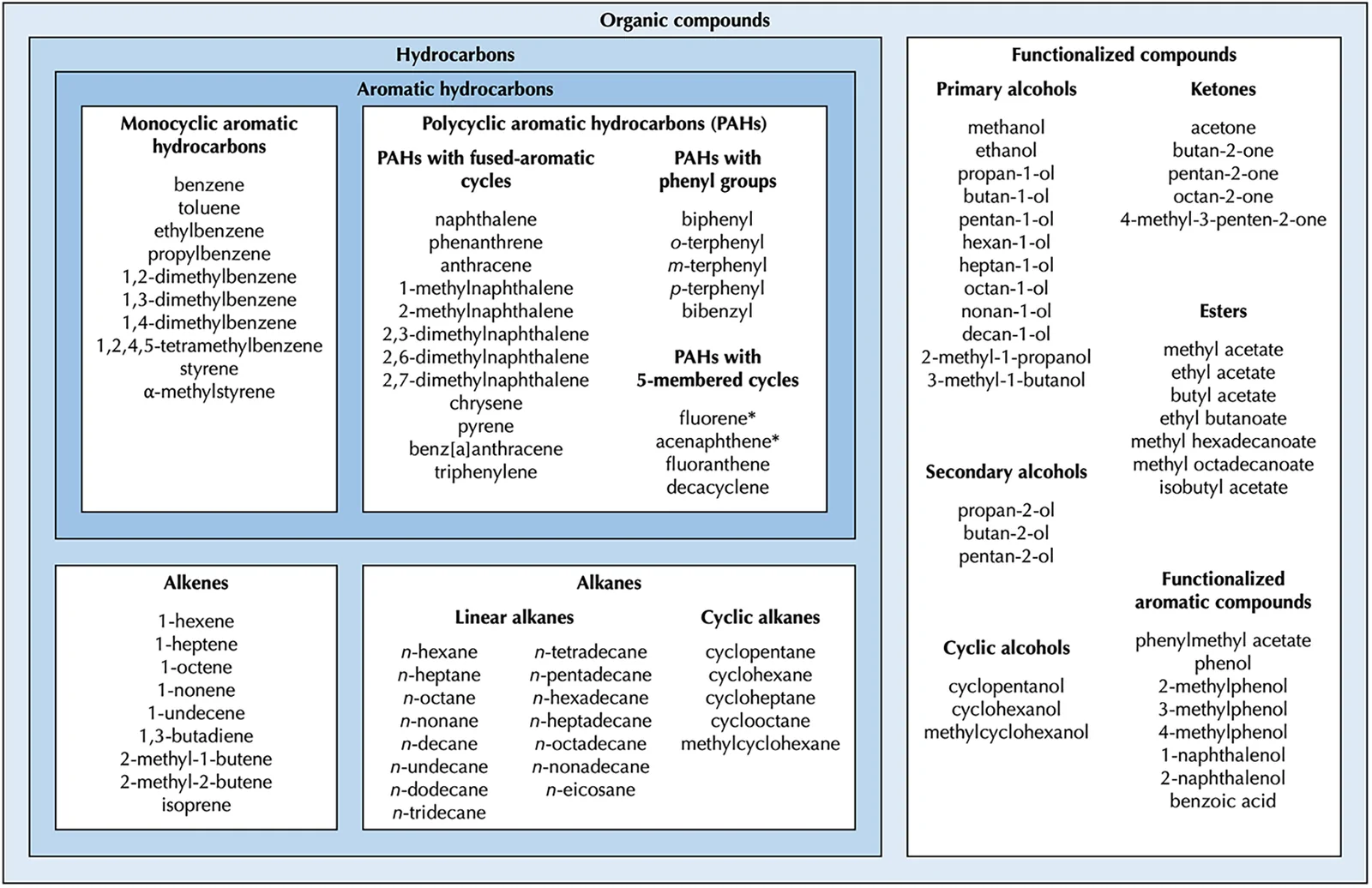
List of compounds considered in our current work, organized by the solvent family. The presence of a partially saturated cycle in a compound is indicated by an asterisk symbol (*).

Descriptions of the SAFT-γ Mie approach, molecular modeling, solubility equations (both for eutectic systems and systems with complete miscibility in the solid phase), and parameter estimation techniques used in the current work are provided in Section 2. Results are detailed in Section 3: new thermodynamic-property calculations for PAHs + aromatic hydrocarbons using recently published parameters^23^ are presented in Section 3.1; results obtained for PAHs + various organic solvents using the new group-interaction parameters characterized in this paper are presented in Section 3.2; and the overall performance of the model is presented and discussed in Section 3.3. Conclusions and additional comments are given in Section 4.

## 2 Methods

### 2.1 SAFT-γ Mie Model

In the SAFT-γ Mie approach,^19,20^ molecules are modeled as associating heteronuclear chains of fused spherical segments that interact through Mie potentials of variable range, while hydrogen bonding and strong polar interactions are treated by embedding square-well association sites on a given segment.^21,22,69^ The segments are defined in accordance with commonly observed chemical functional groups, and it is thus assumed that the properties of a given molecule (or mixture) can be obtained by accounting for the appropriate contributions of the groups. The parameters characterizing any given group are treated as transferable to other molecules and mixtures that contain the same group. The SAFT-γ Mie representations of naphthalene, biphenyl, fluorene, and styrene are shown in Figure 3. The list of aromatic hydrocarbons we consider in this work is shown in Table 1, together with their corresponding group description in the SAFT-γ Mie framework.

**Figure 3:**
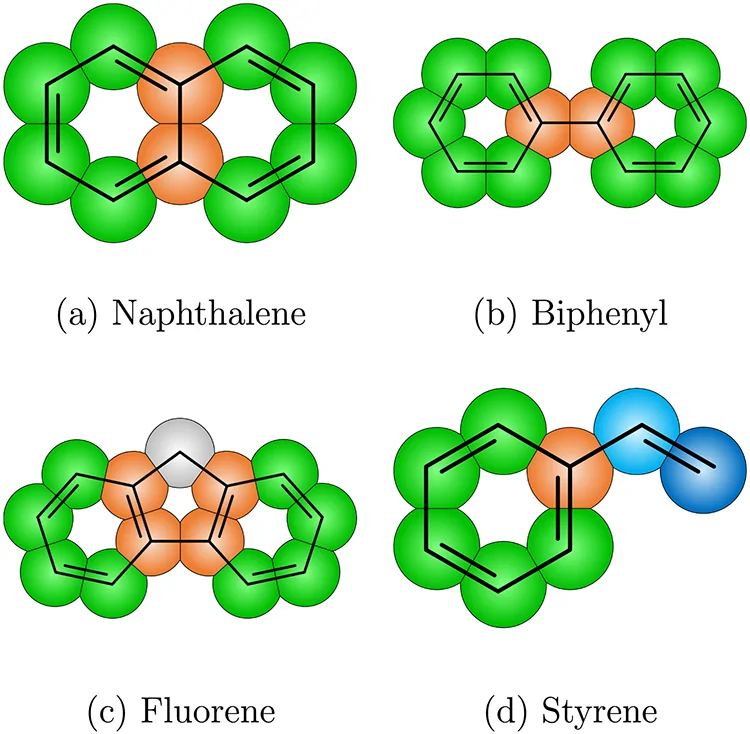
Molecular representation and SAFT-γ Mie molecular model of (a) naphthalene, (b) biphenyl, (c) fluorene, and (d) styrene. The aromatic cycles are modeled with the aCH group (in green) and an aC group (in orange). The aC group is used to represent a fused aromatic carbon (e.g., in naphthalene) or a bridge between an aromatic cycle and other structures (e.g., in biphenyl and styrene). The cCH_2_ group (in gray) is used to model partially saturated aromatic compounds (e.g., in fluorene). The CH= group (in light blue) and the CH_2_= group (in dark blue) are used to model nonaromatic unsaturated bonds (e.g., in the side chain of styrene).

**Table 1:** List of Aromatic Hydrocarbons Considered in Our Current Work, Together with the Corresponding Groups Employed in Modeling the Thermodynamic Properties with SAFT-γ Mie

compound name	CAS number	molecular formula	SAFT-γ Mie modeling
benzene	71-43-2	C_6_H_6_	aCH (×6)
toluene	108-88-3	C_7_H_8_	aCH (×5), aCCH_3_
ethylbenzene	100-41-4	C_8_H_10_	aCH (×5), aCCH_2_, CH_3_
1,2-dimethylbenzene	95-47-6	C_8_H_10_	aCH (×4), aCCH_3_ (×2)
1,3-dimethylbenzene	108-38-3	C_8_H_10_	aCH (×4), aCCH_3_ (×2)
1,4-dimethylbenzene	106-42-3	C_8_H_10_	aCH (×4), aCCH_3_ (×2)
1,2,4,5-tetramethylbenzene	95-93-2	C_10_H_14_	aCH (×2), aCCH_3_ (×4)
styrene	100-42-5	C_8_H_8_	aC, aCH (×5), CH=, CH_2_=
α-methylstyrene	98-83-9	C_9_H_10_	aC, aCH (×5), C=, CH_2_=, CH_3_
naphthalene	91-20-3	C_10_H_8_	aC (×2), aCH (×8)
1-methylnaphthalene	90-12-0	C_11_H_10_	aC (×2), aCH (×7), aCCH_3_
2-methylnaphthalene	91-57-6	C_11_H_10_	aC (×2), aCH (×7), aCCH_3_
2,3-dimethylnaphthalene	581-40-8	C_12_H_12_	aC (×2), aCH (×6), aCCH_3_ (×2)
2,6-dimethylnaphthalene	581-42-0	C_12_H_12_	aC (×2), aCH (×6), aCCH_3_ (×2)
2,7-dimethylnaphthalene	582-16-1	C_12_H_12_	aC (×2), aCH (×6), aCCH_3_ (×2)
phenanthrene	85-01-8	C_14_H_10_	aC (×4), aCH (×10)
anthracene	120-12-7	C_14_H_10_	aC (×4), aCH (×10)
chrysene	218-01-9	C_18_H_12_	aC (×6), aCH (×12)
pyrene	129-00-0	C_16_H_10_	aC (×6), aCH (×10)
benz[*a*]anthracene	56-55-3	C_18_H_12_	aC (×6), aCH (×12)
triphenylene	217-59-4	C_18_H_12_	aC (×6), aCH (×12)
biphenyl	92-52-4	C_12_H_10_	aC (×2), aCH (×10)
*o*-terphenyl	84-15-1	C_18_H_14_	aC (×4), aCH (×14)
*m*-terphenyl	92-06-8	C_18_H_14_	aC (×4), aCH (×14)
*p*-terphenyl	92-94-4	C_18_H_14_	aC (×4), aCH (×14)
bibenzyl	103-29-7	C_14_H_14_	aCH (×10), aCCH_2_ (×2)
fluorene	86-73-7	C_13_H_10_	aC (×4), aCH (×8), cCH_2_
acenaphthene	83-32-9	C_12_H_10_	aC (×4), aCH (×6), cCH_2_ (×2)
fluoranthene	206-44-0	C_16_H_10_	aC (×6), aCH (×10)
decacyclene	191-48-0	C_36_H_18_	aC (×18), aCH (×18)

In addition to aromatic hydrocarbons, several families of organic solvents are modeled in our current work: linear and cyclic alkanes, alkenes, primary and secondary alcohols, acetone and 2-ketones, esters, branched compounds, and functionalized aromatic compounds (e.g., phenol and benzoic acid derivatives). Linear alkanes are modeled with CH_3_ and CH_2_ groups.^19^ Branched compounds are modeled with a CH group.^20^ The cCH_2_ group^20^ is used to model cyclic alkanes, and the cCH group^70^ represents the junction between a nonaromatic cycle and a side chain. Alkenes and other compounds that contain unsaturated nonaromatic bonds are modeled with the CH_2_=, CH=, and C= groups.^20,71^ Methanol is described by the CH_3_OH molecular group.^20^ The CH_2_OH group^33^ is used to model other primary alcohols. Secondary alcohols are modeled with SAFT-γ Mie through the CHOH group^72^ for linear carbon chains, and through the cCHOH group for nonaromatic cycles.^37^ A specific CH_3_COCH_3_ molecular group^20^ is used to model acetone, and a CH_3_CO group^34^ is used to model longer 2-ketones. Esters are modeled with the COO group,^19^ phenols with the aCOH group,^72^ and benzoic acid derivatives with the aCCOOH group.^34^

A given group *k* incorporates a number ν_k_^*^ of identical spherical segments and a shape factor *S*_*k*_ (0 ≤ *S*_*k*_ ≤ 1), which is introduced to characterize the contribution of the segment to the overall thermodynamic properties of the molecule. Interactions between group *k* and group *l* are described by the segment diameter σ_*kl*_, the depth of the potential well ε_*kl*_, and the repulsive and attractive exponents of the Mie potential, denoted by λ_*kl*_^r^ and λ_*kl*_^a^, respectively. Attractive directional interactions are represented by short-ranged square-well association sites placed on the segments. The parameter *N*_ST, *k*_ corresponds to the number of association site types in a group *k*, and *n*_*k*,*a*_ to the number of sites of a given type *a* = 1, ···, *N*_ST, *k*_. The interaction between a site of type *a* placed on a segment of type *k* and a site of type *b* placed on a segment of type *l* is described by an association energy ε_*kl*,*ab*_^HB^ and a bonding volume *K*_*kl*,*ab*_^HB^, which represent the strength and range of the association, respectively. The interactions between groups *k* and *l* involve unlike parameters, which can be obtained through combining rules^19^ (CR) in the first instance. In our current work, however, ε_*kl*_, ε_*kl*,*ab*_^HB^, and *K*_*kl*,*ab*_^HB^ are systematically estimated by comparing target calculated and experimental thermophysical properties of pure fluids or mixtures in which the functional group is present, and λ_*kl*_^r^ is occasionally estimated for better description of the thermodynamic data.

In the SAFT-γ Mie approach, the total Helmholtz free energy *A* of a fluid mixture of nonionic species is expressed as the sum of four contributions^19–22^

1 $$A=A^{\mathrm{ideal}}+A^{\mathrm{monomer}}+A^{\mathrm{chain}}+A^{\mathrm{association}}$$

where *A* ^ideal^ is the ideal free energy of the mixture, *A* ^monomer^ is the contribution accounting for the interactions between Mie segments, *A* ^chain^ is the free energy associated with the formation of chains, and *A* ^association^ is the contribution to the free energy due to association. The Born and ionic contributions, which appear in the complete SAFT-γ Mie free-energy expression,^72^ are not included in eq 1 since we do not consider any ionic species in our current work. Detailed expressions for each contribution can be found in previous work.^19–22,39^ The equilibrium thermodynamic properties of a fluid with *N*_*c*_ components can be determined from the total Helmholtz free energy *A* at a temperature *T*, volume *V*, and vector *N* of the number of molecules, composed of elements *N*_*i*_ of each compound *i*, such that *N* = ∑_*i* = 1_^*N*_*c*_^ *N*_*i*_ is the total number of molecules in the system. The pressure, *P*, and chemical potential of compound *i*, μ_*i*_, are obtained from the standard thermodynamic relations of the Helmholtz free energy *A* and are used to determine the fluid-phase equilibrium conditions.^72^

### 2.2 Solid–Liquid Equilibria

The fusion of a pure compound *i* can be characterized by the melting point *T*_*i*_^fus^, the molar enthalpy of fusion Δ*h*_*i*_^fus^ (*T*_*i*_^fus^, *P*), and the change in heat capacity in going from the solid phase (S) to the liquid phase (L), i.e., Δ*c*_*p*,*i*_ (*T*_*i*_^fus^,*P*) = *c*_*p*,*i*_^L^ (*T*_*i*_^fus^, *P*) – *c*_*p*,*i*_^S^ (*T*_*i*_^fus^, *P*). For a given temperature *T* and pressure *P*, the equilibrium constant of the fusion of a pure compound *i* can be approximated by^34,78^

2 $$K_{i}\left(T,P\right)=\exp\left[-\frac{\Delta h_{i}^{\mathrm{fus}}\left(T_{i}^{\mathrm{fus}},P\right)}{R}\left(\frac{1}{T}-\frac{1}{T_{i}^{\mathrm{fus}}}\right)\;-\frac{\Delta c_{p_{,}i}\left(T_{i}^{\mathrm{fus}},P\right)}{R}\left(\ln\left(\frac{T_{i}^{\mathrm{fus}}}{T}\right)-\frac{T_{i}^{\mathrm{fus}}}{T}+1\right)\right]$$

The values of *T*_*i*_^fus^ and Δ*h*_*i*_^fus^ (*T*_*i*_^fus^, *P*) used in our current work are taken from experimental data^34,73–77^ and are explicitly listed for each compound in Table 2. Δ*c*_*p*,*i*_ (*T*_*i*_^fus^, *P*) is neglected in our calculations due to the lack of experimental data for the compounds considered.

**Table 2:** Melting Point *T*_*i*_^fus^ and Molar Enthalpy of Fusion Δ*h*_*i*_^fus^ (Given at *T*_*i*_^fus^ and Ambient Pressure) of the Compounds Considered in Our Current Work

compound *i*	*T*_*i*_^fus^/K	Δ*h*_*i*_^fus^/ (kJ/mol)	refs	compound *i*	*T*_*i*_^fus^/K	Δ*h*_*i*_^fus^/ (kJ/mol)	refs
benzene	278.325	9.82	^73^	*n*-nonane	219.6	15.6	^74^
toluene	178.2	6.60	^74^	*n*-decane	243.5	28.7	^74^
ethylbenzene	178.2	9.20	^74^	*n*-undecane	247.6	22.1	^74^
1,2-dimethylbenzene	248	13.6	^74^	*n*-dodecane	263.6	35.9	^74^
1,3-dimethylbenzene	225.3	11.6	^74^	*n*-tridecane	267.8	28.5	^74^
1,4-dimethylbenzene	286.4	17.1	^74^	*n*-tetradecane	279	45.6	^74^
1,2,4,5-tetramethylbenzene	352.4	21.0	^74^	*n*-pentadecane	283.1	34.7	^74^
styrene	242.55	11.0	^75^	*n*-hexadecane	291.3	51.8	^74^
α-methylstyrene	249.94	11.9	^75^	*n*-heptadecane	295.1	40.2	^74^
naphthalene	353.5	19.3	^74^	*n*-octadecane	301.3	61.9	^74^
1-methylnaphthalene	242.7	4.90	^74^	*n*-nonadecane	305	45.8	^74^
2-methylnaphthalene	307.7	11.7	^74^	*n*-eicosane	309.6	69.9	^74^
2,3-dimethylnaphthalene	377.15	25.1	^75^	cyclopentane	179.3	0.60	^74^
2,6-dimethylnaphthalene	383.15	24.3	^75^	cyclohexane	279.75	2.63	^75^
2,7-dimethylnaphthalene	369.15	23.3	^75^	cycloheptane	265.12	1.88	^75^
phenanthrene	372.4	16.4	^74^	cyclooctane	287.98	2.41	^75^
anthracene	488.9	28.8	^74^	methanol	175.49	3.21	^76^
chrysene	531.1	26.2	^74^	ethanol	158.65	5.02	^75^
pyrene	423.4	17.4	^74^	propan-1-ol	147.05	5.19	^75^
benz[*a*]anthracene	431.15	21.4	^75^	butan-1-ol	183.35	9.28	^75^
triphenylene	471.2	24.7	^74^	pentan-1-ol	194.25	9.83	^75^
biphenyl	342.4	18.6	^74^	hexan-1-ol	228.6	15.4	^74^
*o*-terphenyl	331.15	17.2	^75^	heptan-1-ol	239.1	18.0	^74^
*m*-terphenyl	362.15	24.1	^75^	octan-1-ol	258.2	21.8	^74^
*p*-terphenyl	486.15	35.5	^75^	nonan-1-ol	268.1	25.7	^74^
bibenzyl	325.15	22.7	^75^	decan-1-ol	280	29.5	^74^
fluorene	389.6	20.1	^74^	phenol	314	11.5	^74^
acenaphthene	366.6	21.5	^74^	2-methylphenol	304.1	13.0	^74^
fluoranthene	380.95	18.9	^75^	3-methylphenol	285.4	9.40	^74^
decacyclene	666	25.4	^77^	4-methylphenol	307.9	11.9	^74^
*n*-hexane	177.8	13.1	^74^	1-naphthalenol	368.5	23.5	^74^
*n*-heptane	182.5	14.1	^74^	2-naphthalenol	395	18.9	^74^
*n*-octane	216.4	20.7	^74^	benzoic acid	395.64	17.9	^34^

For mixtures, we examine the solubility of a compound *i* in a given solvent for two cases: eutectic systems and systems that exhibit complete miscibility in the solid phase. The equilibrium constant can be expressed as

3 $$K_{i}\left(T,P\right)=\frac{a_{i}^{L}}{a_{i}^{S}}$$

where *a*_*i*_^L^ and *a*_*i*_^S^ are the activities of compound *i* in the mixture and, since the reference state of the liquid mixture is pure liquid *i*, the equilibrium constant *K*_*i*_ is also given by eq 2. In the case of eutectic systems, the activity of compound *i* in the saturated solution at a given temperature *T*, pressure *P*, and composition ***x*** ^sat^ is

4 $$a_{i}^{L}\left(T,P,x^{\mathrm{sat}}\right)=\gamma_{i}^{L}\left(T,P,x^{\mathrm{sat}}\right)x_{i}^{S_{i}+L}$$

where γ_*i*_^L^ is the corresponding activity coefficient and *x*_*ii*_^S+L^ is the solubility of compound *i* in the solvent. In the eutectic system, the compounds in the mixture are assumed to be completely immiscible in the solid phase, such that as many solid phases are formed as there are solid components. As a result, the activity of compound *i* can be taken as

5 $$a_{i}^{S}=1$$

The solubility of compound *i* is then $x_{i}^{S_{i}+L}=\frac{K_{i}}{\gamma_{i}^{L}}$ . In a binary mixture, the SLE boundary is given by two curves. For the solubility of solid 1 (written here in terms of mole fraction of component 2), we obtain

6 $$x_{2}^{S_{1}+L}=1-\frac{K_{1}}{\gamma_{1}^{L}}$$

and for the solubility of solid 2, we obtain

7 $$x_{2}^{S_{2}+L}=\frac{K_{2}}{\gamma_{2}^{L}}$$

The two curves intersect at the eutectic point in the *T*–*x*_2_ diagram. A schematic representation of the phase diagram for a eutectic system is shown in Figure 4(a).

**Figure 4:**
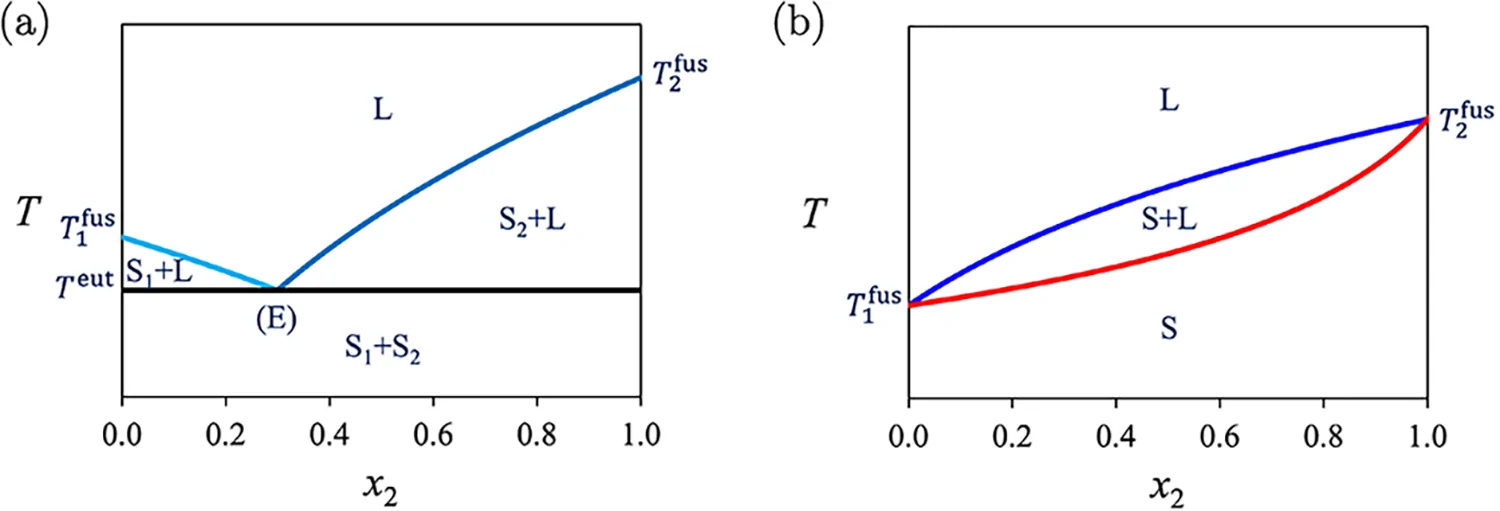
Schematic isobaric phase diagrams with solid–liquid equilibria for a binary mixture. (a) The eutectic system is completely immiscible in the solid phase (S_1_+S_2_). The solubility curves are calculated with eq 6 for compound 1 (light blue) and with eq 7 for compound 2 (dark blue). The two solubility curves intersect at the eutectic point (E), which characterizes the eutectic temperature *T* ^eut^ (black horizontal line). (b) System that is completely miscible in the solid phase (S). The liquidus (blue) is calculated with eq 12 and the solidus (red) is calculated with eq 13.

By contrast, when two compounds are completely miscible in the solid phase, the SLE is characterized by a liquidus and a solidus. From eq 3, the equilibrium constants involved can be expressed as^79^

8 $$K_{1}=\frac{\left(1-x_{2}^{L}\right)\gamma_{1}^{L}}{\left(1-x_{2}^{S}\right)\gamma_{1}^{S}}$$

for solid 1, and

9 $$K_{2}=\frac{x_{2}^{L}\gamma_{2}^{L}}{x_{2}^{S}\gamma_{2}^{S}}$$

for solid 2. From eqs 8 and ([Disp-formula eq9]), the composition of compound 2 can be expressed as

10 $$x_{2}^{L}=\frac{\gamma_{1}^{L}\gamma_{2}^{S}K_{2}-\gamma_{1}^{S}\gamma_{2}^{S}K_{1}K_{2}}{\gamma_{1}^{L}\gamma_{2}^{S}K_{2}-\gamma_{1}^{S}\gamma_{2}^{L}K_{1}}$$

for the liquidus, and as

11 $$x_{2}^{S}=\frac{\gamma_{1}^{L}\gamma_{2}^{L}-\gamma_{1}^{S}\gamma_{2}^{L}K_{1}}{\gamma_{1}^{L}\gamma_{2}^{S}K_{2}-\gamma_{1}^{S}\gamma_{2}^{L}K_{1}}$$

for the solidus. In our work, we consider that the two compounds involved in such systems are ideally miscible in the solid phase (γ_1_^S^ = γ_2_^S^ = 1), such that the liquidus composition is then given as

12 $$x_{2}^{L}=\frac{\gamma_{1}^{L}K_{2}-K_{1}K_{2}}{\gamma_{1}^{L}K_{2}-\gamma_{2}^{L}K_{1}}$$

and the solidus composition as

13 $$x_{2}^{S}=\frac{\gamma_{1}^{L}\gamma_{2}^{L}-\gamma_{2}^{L}K_{1}}{\gamma_{1}^{L}K_{2}-\gamma_{2}^{L}K_{1}}$$

We show a schematic representation of the phase diagram for a system completely miscible in the solid phase in Figure 4(b). As SAFT is a theory for fluid phases and fluid-phase equilibria, we must be informed experimentally if a mixture corresponds to a eutectic system (described by eqs 6 and ([Disp-formula eq7])) or to a system completely miscible in the solid phase (described by eqs 12 and ([Disp-formula eq13])). In both cases, the predictive capability of SAFT concerns the determination of the activity coefficients of the compounds in the liquid phase.

It is worth noting that with the group-contribution method used, the fluid properties will be identical for two different mixtures described with the same SAFT-γ Mie functional groups. However, the SLE obtained for these mixtures must differ since the compounds involved have distinct melting properties (either for eutectic systems or compounds that are completely miscible in the solid phase).

### 2.3 Parameter Estimation

The new group-interaction parameters of SAFT-γ Mie are characterized by minimizing an objective function *f*_obj_, such that

14 $$\min_{\Theta }f_{\mathrm{obj}}=\frac{1}{N^{\exp}}\sum_{s=1}^{N^{S}}\sum_{p=1}^{N_{s}^{P}}\sum_{i=1}^{N_{s,p}^{D}}w_{s,p,i}{\left(\frac{X_{s,p,i}^{\exp}-X_{s,p,i}^{\mathrm{calc}}\left(\Theta \right)}{X_{s,p,i}^{\exp}}\right)}^{2}$$

where Θ is the vector of model parameters, *N* ^exp^ the number of experimental points considered in the parameter estimation, *N* ^S^ the number of systems (pure compounds or binary mixtures) used in the estimation, *N*_*s*_^P^ the number of properties for system *s*, *N*_*s*, *p*_^D^ the number of experimental data points for system *s* and property *p*, and *w*_*s*,*p*,*i*_ a weight that controls the relative importance of data point *i* for property *p* of system *s* (we use an equal weight *w*_*s*,*p*,*i*_ = 1 in the current work). *X*_*s*,*p*,*i*_^exp^ is the *i*th measured value in the property *p* data vector of system *s*, and *X*_*s*, *p*,*i*_^calc^(Θ) is the *i*th calculated value for property *p* and system *s* using the SAFT-γ Mie approach and the parameters Θ.

To quantitatively assess the accuracy of the results obtained with SAFT-γ Mie for a system *s* and property *p*, we define the percentage absolute average deviation (%AAD) as

15 $$\%{\mathrm{AAD}}_{s}\left[p\right]=\frac{1}{N_{s,p}^{D}}\sum_{i=1}^{N_{s,p}^{D}}\left|\frac{X_{s,p,i}^{\exp}-X_{s,p,i}^{\mathrm{calc}}}{X_{s,p,i}^{\exp}}\right|\times 100$$

and the average absolute deviation (AAD) as

16 $${\mathrm{AAD}}_{s}\left[p\right]=\frac{1}{N_{s,p}^{D}}\sum_{i=1}^{N_{s,p}^{D}}\left|X_{s,p,i}^{\exp}-X_{s,p,i}^{\mathrm{calc}}\right|$$

AADs are more relevant than %AADs to assess the quality of solubility predictions due to the orders of magnitude involved in the solubility values.

We use the gPROMS^80^ tools to perform parameter estimation and calculations in our current work. These calculations can also be reproduced with open-source toolkits, such as SGTPy^81^ and Clapeyron.jl.^82^

## 3 Results

The list of the group interactions fully characterized in previous studies^19,20,23,33,34,36,37,42,70–72,83–85^ and in our current work is summarized in Table 3. The parameters characterized in previous works for the aCH group^20^ and the aC group^23^ (employed in the molecular modeling of all the PAHs considered) are used in Section 3.1 to assess the capability of SAFT-γ Mie to model phenyls and PAHs with 5-membered rings and to predict the SLE of binary mixtures that contain PAHs (which have not been considered in previous work with SAFT-γ Mie). The new SAFT-γ Mie parameters developed in our current work are summarized in Table 4 (more information about the parameter estimation and the order of the fitting procedure can be found in a Zenodo repository file; please see the Data Availability Statement). We note that the values of ε_*kl*_ obtained for unlike interactions that involve the aC group can be large (e.g., for the aC–CH, aC–CHOH, and aC–COO interactions) to compensate the small values reported for the like interactions of the aC group (e.g., ε_aC–aC_/*k*_B_ = 80.000 K and *S*_aC_ = 0.018000) in previous work.^23^ By contrast, the values of ε_*kl*_ can tend to 0.0000 to compensate for large values of like interactions (e.g., for the C= group). The new parameters are used to study mixtures of aromatic compounds in various organic solvents, as presented in Section 3.2. The overall quality of the approach to predict the SLE of systems composed of PAHs is discussed in Section 3.3.

**Table 3:**
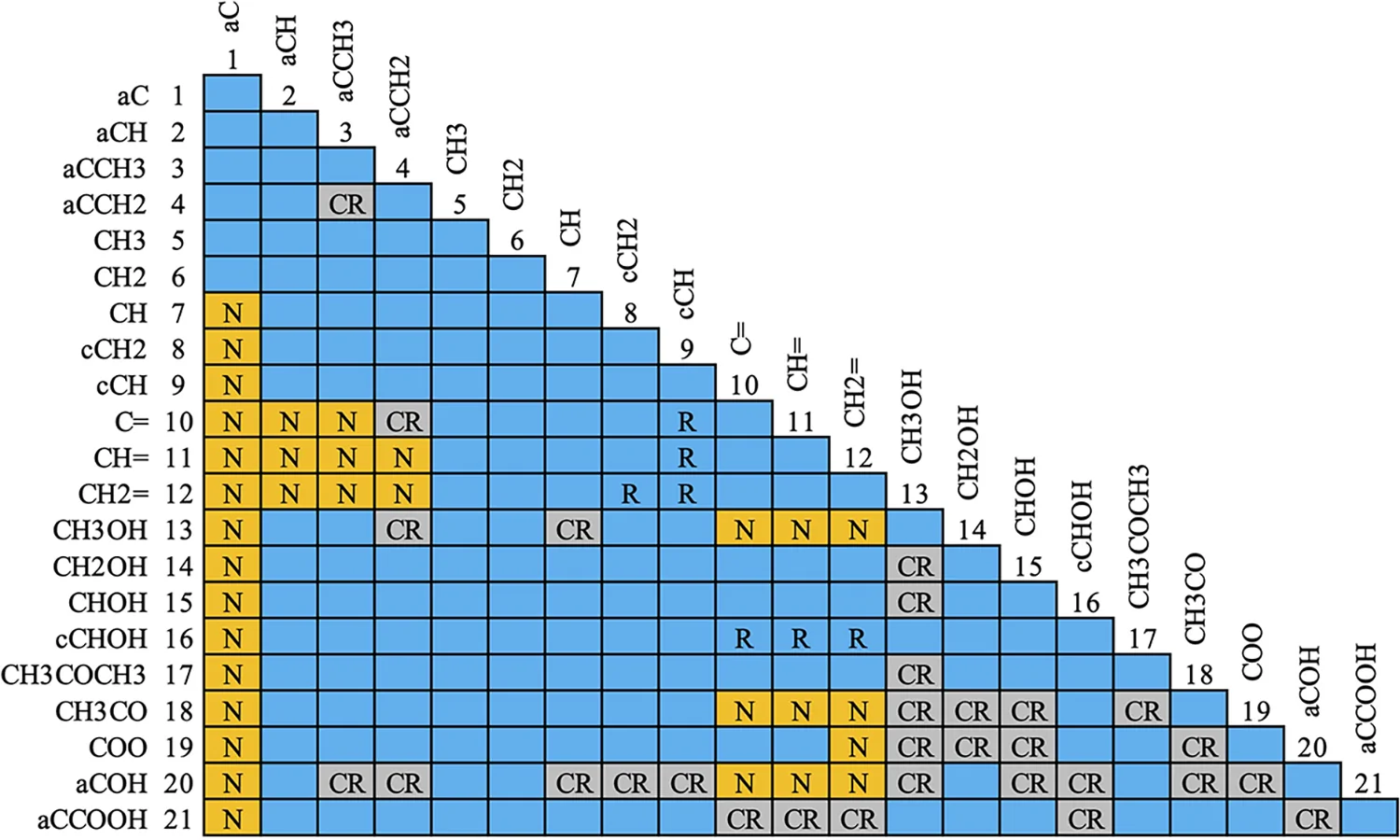
Summary of the Group Interactions Used to Model the Systems (Pure Fluids or Mixtures) Considered in Our Current Work with the SAFT-γ Mie Approach^a^

a Blue shading denotes that group-interaction parameters have been optimized in previous publications^19,20,23,33,34,36,37,70–72,83–85^ and recent (R) work.^42^ The new (N) parameters optimized in our current work are denoted with yellow shading. Gray shading denotes that unlike interaction parameters are predicted using combining rules (CR).^19^

**Table 4:** New SAFT-γ Mie Unlike Group Parameters Characterized in Our Current (*) and Recent^42^ Work^a^

*k*	*l*	group *k*	group *l*	(ε_*kl*_/*k*_B_)/K	λ_*kl*_^r^	refs
1	7	aC	CH	1771.5	CR	*
1	8	aC	cCH_2_	1208.2	CR	*
1	9	aC	cCH	1000.0	CR	*
1	10	aC	C=	0.0000	CR	*
1	11	aC	CH=	750.00	CR	*
1	12	aC	CH_2_=	1000.0	CR	*
1	13	aC	CH_3_OH	559.39	8.0000	*
1	14	aC	CH_2_OH	959.86	CR	*
1	15	aC	CHOH	1829.2	CR	*
1	16	aC	cCHOH	900.00	CR	*
1	17	aC	CH_3_COCH_3_	611.60	8.0000	*
1	18	aC	CH_3_CO	1029.9	CR	*
1	19	aC	COO	1423.7	CR	*
1	20	aC	aCOH	708.12	CR	*
1	21	aC	aCCOOH	763.08	CR	*
2	10	aCH	C=	0.0000	CR	*
2	11	aCH	CH=	138.00	CR	*
2	12	aCH	CH_2_=	507.30	CR	*
3	10	aCCH_3_	C=	954.09	CR	*
3	11	aCCH_3_	CH=	770.00	CR	*
3	12	aCCH_3_	CH_2_=	251.00	CR	*
4	11	aCCH_2_	CH=	747.67	CR	*
4	12	aCCH_2_	CH_2_=	415.73	CR	*
8	12	cCH_2_	CH_2_=	417.00	CR	^42^
9	10	cCH	C=	0.0000	CR	^42^
9	11	cCH	CH=	144.00	CR	^42^
9	12	cCH	CH_2_=	144.00	CR	^42^
10	13	C=	CH_3_OH	325.09	CR	*
10	16	C=	cCHOH	0.0000	CR	^42^
10	18	C=	CH_3_CO	700.00	CR	*
10	20	C=	aCOH	793.91	CR	*
11	13	CH=	CH_3_OH	394.15	CR	*
11	16	CH=	cCHOH	538.32	CR	^42^
11	18	CH=	CH_3_CO	100.00	CR	*
11	20	CH=	aCOH	612.45	CR	*
12	13	CH_2_=	CH_3_OH	325.00	CR	*
12	16	CH_2_=	cCHOH	0.0000	CR	^42^
12	18	CH_2_=	CH_3_CO	575.00	CR	*
12	19	CH_2_=	COO	380.00	CR	*
12	20	CH_2_=	aCOH	351.64	CR	*

a The use of combining rules for λ_kl_^r^ is denoted as CR (cf. Equation (76) in Papaioannou et al.^19^).

### 3.1 SAFT-γ Mie Phase Equilibrium Property Predictions for PAHs + Aromatic Hydrocarbon Mixtures

We provide examples of SLE prediction with the SAFT-γ Mie approach for PAHs + aromatic hydrocarbon mixtures, for which extensive experimental data are available^15,16,55,86–144^ (only selected examples are presented here). All AADs for SLE composition are summarized in Table 5 in Section 3.3. The AADs for other thermodynamic properties are provided in Table 6 in Section 3.3.

**Table 5:** Overview of Accuracy of SAFT-γ Mie in the Calculation of Mole Fraction Solubility *x* ^sat^ of PAHs + Organic Solvents^a^

system *s* (1 + 2)	*T*/K	*N*_*s*,*p*_^est^	*N*_*s*,*p*_^tot^	AAD	refs	Figures
naphthalene + phenanthrene	364–322–350	0	45	0.0176	^15,86,87^	[Fig fig5](c), 12
naphthalene + anthracene	479–349–352	0	38	0.0358	^87–89^	[Fig fig5](c), 12
naphthalene + biphenyl	337–312–348	0	39	0.0160	^87,90–92^	[Fig fig6](d), 12
naphthalene + chrysene	512–351–351	0	10	0.0372	^87^	[Fig fig12]
naphthalene + pyrene	409–339–350	0	12	0.0319	^87^	[Fig fig12]
naphthalene + *o*-terphenyl	325–305	0	2	0.0227	^93^	[Fig fig6](d), 12
naphthalene + *m*-terphenyl	348–323	0	2	0.0235	^93^	[Fig fig6](d), 12
naphthalene + bibenzyl	320–306–341	0	12	0.0189	^92^	[Fig fig12]
naphthalene + fluoranthene	374–329–350	0	19	0.0282	^87,94^	[Fig fig12]
naphthalene + decacyclene	485–353	0	5	0.178	^95^	N/A
naphthalene + fluorene	378–328–348	0	22	0.0502	^87,96^	[Fig fig12]
naphthalene + acenaphthene	362–325–349	0	41	0.0255	^87,97,98^	[Fig fig12]
naphthalene + benzene	276–270–346	0	18	0.0132	^99^	[Fig fig5](a), 12
naphthalene + toluene	178–177–346	0	21	0.0350	^100^	[Fig fig5](a), 12
naphthalene + ethylbenzene	178–178–344	0	15	0.0128	^101^	[Fig fig5](a), 12
naphthalene + 1,2-dimethylbenzene	246–245–346	0	15	0.0432	^101^	[Fig fig5](b)
naphthalene + 1,3-dimethylbenzene	225–224–346	0	17	0.0517	^102^	[Fig fig5](b)
naphthalene + 1,4-dimethylbenzene	284–277–347	0	13	0.0371	^101^	[Fig fig5](b), 12
naphthalene + 1,2,4,5-tetramethylbenzene	349–321	0	17	0.0318	^103,104^	N/A
naphthalene + 2,3-dimethylnaphthalene	370–327–349	0	22	0.0322	^87,98^	N/A
naphthalene + 2,6-dimethylnaphthalene	377–331–349	0	33	0.0123	^87,98,105^	[Fig fig12]
naphthalene + 2,7-dimethylnaphthalene	365–326–349	0	27	0.0137	^98,105^	N/A
naphthalene + *n*-hexane	282–346	0	49	0.0514	^99,177,178^	N/A
naphthalene + *n*-heptane	273–350	0	14	0.0334	^172,179^	[Fig fig9](a), 12
naphthalene + *n*-octane	293–303	0	3	0.0836	^180–182^	N/A
naphthalene + *n*-decane	303	0	1	0.105	^182^	N/A
naphthalene + *n*-dodecane	284–328	0	12	0.115	^182,183^	N/A
naphthalene + *n*-hexadecane	298–303	0	2	0.137	^182,184^	N/A
naphthalene + *n*-nonadecane	312–348	0	8	0.0295	^185^	N/A
naphthalene + *n*-eicosane	309–308–353	0	37	0.0927	^56,186,187^	[Fig fig9](a)
naphthalene + cyclopentane	298	0	1	0.0144	^188^	[Fig fig8](d)
naphthalene + cyclohexane	271–350	0	28	0.0172	^99,178^	[Fig fig8](d), 12
naphthalene + methanol	273–347	20	20	0.0476	^114,177^	[Fig fig9](b), 12
naphthalene + ethanol	289–347	0	43	0.0624	^99,189,190^	[Fig fig9](b), 12
naphthalene + propan-1-ol	273–343	0	15	0.0401	^114,190^	[Fig fig9](b)
naphthalene + butan-1-ol	284–349	0	43	0.0490	^177,178,189^	[Fig fig9](b)
naphthalene + pentan-1-ol	298	0	1	0.0497	^191^	[Fig fig9](b)
naphthalene + hexan-1-ol	282–332	0	11	0.0700	^178^	[Fig fig9](b)
naphthalene + octan-1-ol	281–332	0	41	0.0894	^178,183,192^	[Fig fig9](b)
naphthalene + nonan-1-ol	293–351	0	11	0.0610	^193^	[Fig fig9](b)
naphthalene + decan-1-ol	303	0	1	0.0984	^182^	[Fig fig9](b)
naphthalene + propan-2-ol	298–343	11	11	0.0333	^190^	[Fig fig9](c), 12
naphthalene + butan-2-ol	302–342	8	8	0.0244	^190^	[Fig fig9](c)
naphthalene + cyclohexanol	283–323	6	6	0.0387	^194,195^	N/A
naphthalene + methylcyclohexanol	283–318	0	5	0.0460	^194^	N/A
naphthalene + acetone	241–343	12	51	0.0563	^177,189,196,197^	[Fig fig9](d), 12
naphthalene + butanone	277–347	0	6	0.0511	^172^	[Fig fig9](d), 12
naphthalene + octan-2-one	281–325	0	11	0.00863	^183^	[Fig fig9](d)
naphthalene + ethyl acetate	278–323	10	10	0.00659	^196^	[Fig fig10](a), 12
naphthalene + methyl hexadecanoate	301–296–350	0	19	0.0358	^198^	[Fig fig10](a)
naphthalene + methyl octadecanoate	312–306–351	0	18	0.111	^198^	[Fig fig10](a)
naphthalene + isobutyl acetate	278–323	0	10	0.00315	^196^	[Fig fig10](b)
naphthalene +2-methyl-1-propanol	293–343	4	9	0.0473	^190^	[Fig fig10](b), 12
naphthalene + phenol	310–303–350	10	12	0.0506	^199^	[Fig fig10](c), 12
naphthalene + 1-naphthalenol	367–334–352	0	50	0.0552	^200–203^	N/A
naphthalene + benzoic acid	388–341–350	5	22	0.0150	^204,205^	[Fig fig10](d), 12
phenanthrene + anthracene (liquidus)	486–376	0	49	0.0885	^15,87,106,107^	[Fig fig12]
phenanthrene + anthracene (solidus)	486–376	0	38	0.0345	^15,87,106,107^	[Fig fig12]
phenanthrene + biphenyl	338–331–365	0	12	0.0801	^87^	[Fig fig12]
phenanthrene + chrysene	515–369–371	0	15	0.0258	^87,107^	[Fig fig12]
phenanthrene + pyrene	413–355–366	0	11	0.0389	^87^	[Fig fig12]
phenanthrene + *o*-terphenyl	312–316	0	2	0.0771	^93^	[Fig fig12]
phenanthrene + *m*-terphenyl	338–330	0	2	0.0282	^93^	[Fig fig12]
phenanthrene + fluoranthene	377–346–369	0	32	0.0784	^87,97,108^	[Fig fig7], 12
phenanthrene + decacyclene	611–373	0	11	0.176	^95^	N/A
phenanthrene + fluorene	384–369–372	0	31	0.195	^87,97,108^	[Fig fig12]
phenanthrene + acenaphthene	365–327–364	0	37	0.0316	^87,108,109^	[Fig fig12]
phenanthrene + benzene	278–342	0	17	0.119	^110–112^	[Fig fig12]
phenanthrene + toluene	273–351	0	29	0.163	^55,113–115^	[Fig fig12]
phenanthrene + 1,2,4,5-tetramethylbenzene	328	0	1	0.158	^104^	N/A
phenanthrene + 1-methylnaphthalene	241–368	0	15	0.0124	^15^	N/A
phenanthrene + 2,6-dimethylnaphthalene	379–344–367	0	9	0.0490	^117^	[Fig fig12]
phenanthrene + 2,7-dimethylnaphthalene	363–331–365	0	13	0.0202	^15^	N/A
phenanthrene + *n*-heptane	293–323	0	5	0.220	^55,143^	[Fig fig12]
phenanthrene + cyclohexane	286–342	0	22	0.141	^112,143,206,207^	[Fig fig12]
phenanthrene + cyclooctane	298	0	1	0.0262	^143^	N/A
phenanthrene + methylcyclohexane	298	1	1	0.0035	^143^	N/A
phenanthrene + methanol	268–303	0	8	0.00796	^111^	[Fig fig12]
phenanthrene + ethanol	263–343	0	19	0.0159	^111,114,172^	[Fig fig12]
phenanthrene + propan-1-ol	298	0	2	0.0520	^143,208^	N/A
phenanthrene + butan-1-ol	298	0	2	0.0923	^143,208^	N/A
phenanthrene + pentan-1-ol	298	0	1	0.126	^143^	N/A
phenanthrene + hexan-1-ol	298	0	1	0.156	^143^	N/A
phenanthrene + heptan-1-ol	298	0	1	0.179	^143^	N/A
phenanthrene + octan-1-ol	277–298	0	5	0.172	^143,209,210^	N/A
phenanthrene + propan-2-ol	298	0	2	0.0172	^143,208^	[Fig fig12]
phenanthrene + butan-2-ol	298	0	1	0.0654	^143^	N/A
phenanthrene + pentan-2-ol	298	0	1	0.116	^143^	N/A
phenanthrene + cyclopentanol	298	1	1	0.0867	^143^	N/A
phenanthrene + acetone	263–308	0	10	0.0130	^111,211^	[Fig fig12]
phenanthrene + butanone	298–327	0	11	0.0785	^143,172^	[Fig fig12]
phenanthrene + ethyl acetate	298	0	2	0.129	^143,210^	[Fig fig12]
phenanthrene + butyl acetate	298	0	1	0.174	^143^	N/A
phenanthrene + 2-methyl-1-propanol	298	0	1	0.0416	^143^	[Fig fig12]
phenanthrene + 3-methyl-1-butanol	298	0	1	0.0699	^143^	N/A
phenanthrene + phenol	323–350	0	8	0.0527	^172^	[Fig fig10](c), 12
anthracene + biphenyl	419–453	0	3	0.0529	^118^	[Fig fig12]
anthracene + chrysene	515–465–485	0	17	0.0430	^87,107^	[Fig fig12]
anthracene + pyrene	419–405–485	0	22	0.0294	^87,119^	[Fig fig12]
anthracene + fluoranthene	378–374–482	0	22	0.00952	^87,94^	[Fig fig7], 12
anthracene + decacyclene	613–487–489	0	10	0.114	^95^	N/A
anthracene + fluorene (eutectic†^b^)	388–378–490	0	16	0.0543	^120^	[Fig fig12]
anthracene + fluorene (liquidus†^b^)	387–489	0	15	0.1081	^15^	N/A
anthracene + fluorene (solidus†^b^)	387–489	0	18	N/A	^15^	N/A
anthracene + acenaphthene (eutectic†^b^)	363–360–482	0	13	0.0464	^120^	[Fig fig12]
anthracene + acenaphthene (liquidus†^b^)	389–485	0	11	0.0230	^317^	N/A
anthracene + acenaphthene (solidus†^b^)	376–453	0	11	0.0361	^317^	N/A
anthracene + benzene	273–378	0	26	0.0422	^110,121^	[Fig fig12]
anthracene + toluene	293–363	0	17	0.0479	^122,123^	[Fig fig12]
anthracene + ethylbenzene	298	0	1	0.0145	^124^	[Fig fig12]
anthracene + 1,2-dimethylbenzene	293–363	0	5	0.0693	^123,125,126^	N/A
anthracene + 1,3-dimethylbenzene	293–393	0	6	0.0895	^123,125,126^	N/A
anthracene + 1,4-dimethylbenzene	293–393	0	6	0.0913	^123,125,127^	[Fig fig12]
anthracene + 2-methylnaphthalene	307–307–488	0	43	0.0358	^89,116^	N/A
anthracene + 2,6-dimethylnaphthalene	379–375–480	0	10	0.0171	^89^	[Fig fig12]
anthracene + 2,7-dimethylnaphthalene	367–364–477	0	10	0.0133	^89^	N/A
anthracene + *n*-heptane	293–352	0	10	0.00977	^122,172^	[Fig fig12]
anthracene + cyclohexane	293–402	0	18	0.0143	^123,212–214^	[Fig fig12]
anthracene + cyclooctane	298	0	1	0.000311	^215^	N/A
anthracene + methanol	298–333	0	6	0.000803	^176^	[Fig fig12]
anthracene + ethanol	286–443	0	42	0.00925	^121,123,172,176,216,217^	[Fig fig12]
anthracene + propan-2-ol	298–333	0	11	0.000957	^176,218^	[Fig fig12]
anthracene + butan-2-ol	298	0	1	0.00197	^219^	N/A
anthracene + pentan-2-ol	298	0	2	0.00329	^220,221^	N/A
anthracene + cyclopentanol	298	1	1	0.00294	^126^	N/A
anthracene + acetone	293–413	0	23	0.0128	^121,123,176,210,222^	[Fig fig12]
anthracene + butanone	287–399	0	11	0.00534	^172,218^	[Fig fig12]
anthracene + methyl acetate	298	0	1	0.00140	^223^	N/A
anthracene + ethyl acetate	298	0	1	0.00588	^224^	[Fig fig12]
anthracene + butyl acetate	298	0	1	0.0149	^214^	N/A
anthracene + 2-methyl-1-propanol	298	0	1	0.00124	^223^	[Fig fig12]
anthracene + 3-methyl-1-butanol	298	0	1	0.00219	^223^	N/A
anthracene + phenol	331–363	0	4	0.00550	^172^	[Fig fig10](c), 12
anthracene + 2-naphthalenol	390–387–473	0	11	0.0980	^88^	N/A
anthracene + benzoic acid	390–392	0	3	0.0390	^225^	[Fig fig12]
biphenyl + *o*-terphenyl	319–303	0	2	0.0124	^93^	[Fig fig12]
biphenyl + *m*-terphenyl	338–319	0	2	0.00587	^93^	[Fig fig12]
biphenyl + *p*-terphenyl	433–341	0	11	0.00705	^128^	[Fig fig12]
biphenyl + bibenzyl	324–303–337	0	40	0.0210	^92,129^	[Fig fig12]
biphenyl + fluorene	379–341	0	9	0.0760	^87^	[Fig fig12]
biphenyl + acenaphthene	361–319–336	0	12	0.0412	^87^	[Fig fig12]
biphenyl + benzene	277–269–336	0	33	0.0186	^130–134^	[Fig fig6](c), 12
biphenyl + 1,2,4,5-tetramethylbenzene	344–314–336	0	10	0.0681	^135^	N/A
biphenyl + 1-methylnaphthalene	240–235–336	0	13	0.0374	^87^	N/A
biphenyl + 2-methylnaphthalene	308–300–337	0	31	0.0727	^87,136^	N/A
biphenyl + 2,3-dimethylnaphthalene	370–329–338	0	12	0.0583	^87^	N/A
biphenyl + 2,6-dimethylnaphthalene	377–324–336	0	12	0.0237	^87^	[Fig fig12]
biphenyl + 2,7-dimethylnaphthalene	364–322–337	0	12	0.0135	^87^	N/A
biphenyl + *n*-hexane	283–341	0	10	0.115	^226–228^	N/A
biphenyl + *n*-heptane	283–341	0	29	0.103	^132,227–229^	[Fig fig12]
biphenyl + *n*-octane	298–313	0	4	0.135	^228^	N/A
biphenyl + *n*-nonane	298	0	1	0.124	^230^	N/A
biphenyl + *n*-decane	298	0	1	0.132	^230^	N/A
biphenyl + *n*-dodecane	261	0	1	0.0314	^231^	N/A
biphenyl + *n*-tridecane	268–265–329	0	10	0.0340	^232^	N/A
biphenyl + *n*-tetradecane	277	0	1	0.0242	^233^	N/A
biphenyl + *n*-hexadecane	298	0	1	0.0802	^230^	N/A
biphenyl + *n*-octadecane	300–299–339	0	24	0.138	^234,235^	N/A
biphenyl + *n*-eicosane	309–309–339	0	10	0.191	^235^	N/A
biphenyl + cyclohexane	273–270–331	0	16	0.00524	^130,206,228^	[Fig fig12]
biphenyl + methanol	293–298	0	6	0.00836	^230,236–238^	[Fig fig12]
biphenyl + ethanol	273–331	0	18	0.0174	^172,227,230,236,237^	[Fig fig12]
biphenyl + propan-2-ol	298	0	1	0.00278	^230^	[Fig fig12]
biphenyl + acetone	298	0	2	0.0658	^237^	[Fig fig12]
biphenyl + methyl hexadecanoate	302–297–340	10	19	0.0609	^198^	N/A
biphenyl + methyl octadecanoate	311–305–339	11	19	0.0657	^198^	N/A
biphenyl + 2-methyl-1-propanol	298	0	1	0.0398	^230^	[Fig fig12]
biphenyl + 3-methyl-1-butanol	298	0	1	0.0502	^230^	N/A
chrysene + pyrene	411–406–514	0	13	0.0356	^87^	[Fig fig5](d)
chrysene + benz[*a*]anthracene (liquidus)	448–525	0	20	0.0156	^16,87^	[Fig fig5](d)
chrysene + benz[*a*]anthracene (solidus)	448–525	0	9	0.0601	^16,87^	[Fig fig5](d)
chrysene + fluoranthene	380–517	0	10	0.0187	^87^	N/A
chrysene + benzene	298–345	0	6	0.0517	^133,134,137^	N/A
chrysene + toluene	291–373	0	2	0.119	^138^	N/A
chrysene + butanone	291–383	0	7	0.0204	^172^	N/A
chrysene + phenol	336–391	0	4	0.0230	^172^	N/A
pyrene + fluoranthene	374–368–413	0	9	0.0876	^87^	N/A
pyrene + benzene	293–358	0	13	0.175	^133,134,137,139^	N/A
pyrene + toluene	293–333	0	14	0.256	^55,140–142^	N/A
pyrene + ethylbenzene	293–318	0	7	0.151	^139,143^	N/A
pyrene + 1,2-dimethylbenzene	299	0	1	0.301	^141^	N/A
pyrene + 1,3-dimethylbenzene	299	0	1	0.307	^141^	N/A
pyrene + 1,4-dimethylbenzene	293–318	0	7	0.293	^141,144^	N/A
pyrene + 2-methylnaphthalene	306–300–422	0	33	0.105	^116^	N/A
pyrene + methylcyclohexane	293–318	7	7	0.00284	^139,239^	N/A
pyrene + cyclopentanol	299	1	1	0.110	^141^	N/A
pyrene + cyclohexanol	299	1	1	0.105	^240^	N/A
pyrene + butanone	299	0	1	0.0310	^143^	N/A
pyrene + methyl acetate	299	0	1	0.0520	^241^	N/A
pyrene + ethyl acetate	295–333	0	10	0.152	^140,141^	N/A
pyrene + butyl acetate	299	0	1	0.202	^141^	N/A
pyrene + ethyl butanoate	294	0	1	0.192	^142^	N/A
pyrene + 2-methyl-1-propanol	299	0	1	0.0270	^141^	N/A
pyrene + 3-methyl-1-butanol	299	0	1	0.0547	^141^	N/A
triphenylene + benzene	313–356	0	5	0.121	^134^	N/A
triphenylene + benz[*a*]anthracene	427–404–457	0	10	0.0481	^16^	N/A
*o*-terphenyl + benzene	301–324	0	4	0.0240	^134^	[Fig fig6](c)
*m*-terphenyl + benzene	310–351	0	6	0.0269	^134^	[Fig fig6](c)
*p*-terphenyl + benzene	311–351	0	5	0.0188	^134^	[Fig fig6](c)

a *N*_*s*,*p*_^est^ is the number of experimental data used in parameter estimation, and *N*_*s*,*p*_^tot^ is the number of experimental data used to calculate the AAD_*s*_ [*p*], for system *s* and property *p*. Two values (*T*′–*T*″) are mentioned to define the temperature range if no eutectic point is found in the experimental data. Three values (*T*′–*T*_min_–*T*″) are mentioned to define the temperature range if a eutectic point is found in the experimental data; the solubility of compound 2 is given from *T*′ to *T*_min_, and the solubility of compound 1 is given from *T*_min_ to *T*″(the presence of *T*_min_ reveals the presence of a eutectic but is not necessarily the eutectic temperature). For a system that is completely miscible in the solid phase, the accuracy is specified both for the liquidus and the solidus.

b The dagger indicates that several representations are found in the literature for a given mixture (eutectic and completely miscible in the solid phase).

**Table 6:** Overview of Accuracy of SAFT-γ Mie in the Calculation of the Vapor Pressure, Liquid Density of the Pure Fluid, Saturation (sat.) Density, Vaporization Enthalpy, Bubble (bub.) and Dew Temperature and Pressure, Density of Binary Mixtures, and Excess Enthalpy of Aromatic Compounds + Organic Solvents^a^

Vapor Pressure									
system *s* (pure)	*T*/K	*P*/kPa	*x*_1_	*N*_*s*,*p*_^est^	*N*_*s*,*p*_^tot^	%AAD	AAD/kPa	refs	Figures
biphenyl	345–700	sat.	pure	0	72	29.43	73.72	^150^	[Fig fig6](a)
fluorene	425–639	sat.	pure	0	45	25.19	16.12	^151–153^	[Fig fig8](a)
acenaphthene	363–561	sat.	pure	0	52	10.48	3.830	^151,154,155^	[Fig fig8](a)
fluoranthene	383–673	sat.	pure	0	45	29.91	5.475	^156,157^	N/A
1-naphthalenol	399–556	sat.	pure	9	9	23.78	3.440	^158^	[Fig fig11](c)
2-naphthalenol	402–561	sat.	pure	0	9	12.50	0.7672	^158^	[Fig fig11](c)
styrene	246–635	sat.	pure	111	111	3.720	10.27	^242–249^	N/A
α-methylstyrene	274–438	sat.	pure	22	22	1.743	0.5856	^249–251^	N/A
4-methyl-3-penten-2-one	273–442	sat.	pure	26	26	4.402	1.5923	^252–254^	N/A

Liquid Density									
system *s* (pure)	*T*/K	*P*/kPa	*x*_1_	*N*_*s*,*p*_^est^	*N*_*s*,*p*_^tot^	%AAD	AAD/(kg/m^3^)	refs	Figures
biphenyl	345–700	sat.	pure	0	72	2.307	18.20	^150^	[Fig fig6](b)
fluorene	293–386	101.3	pure	0	2	5.441	61.27	^159,160^	[Fig fig8](b)
acenaphthene	373–773	sat.	pure	0	11	7.710	59.79	^148^	[Fig fig8](b)
fluoranthene	393–843	sat.	pure	0	11	9.097	79.14	^148^	N/A
1-naphthalenol	374–408	101.3	pure	0	3	1.205	13.13	^161^	N/A
2-naphthalenol	403–434	101.3	pure	0	3	1.322	14.11	^161^	N/A
styrene	273–418	101.3	pure	0	49	1.538	13.46	^255–258^	N/A
α-methylstyrene	293–373	101.3	pure	0	12	5.072	45.01	^259–261^	N/A
4-methyl-3-penten-2-one	273–393	101.3	pure	0	10	0.5402	4.288	^252,260^	N/A

Vaporization Enthalpy									
system *s* (pure)	*T*/K	*P*	*x*_1_	*N*_*s*,*p*_^est^	*N*_*s*,*p*_^tot^	%AAD	AAD/(kJ/mol)	refs	Figures
biphenyl	345–700	sat.	pure	0	72	6.263	2.790	^150^	N/A
fluorene	298–399	sat.	pure	0	4	2.665	1.804	^160,162–164^	[Fig fig8](c)
acenaphthene	373–723	sat.	pure	0	11	1.160	0.5683	^148^	[Fig fig8](c)
fluoranthene	383–893	sat.	pure	0	43	8.118	4.970	^148,157^	N/A

*Tx* Diagram									
system *s* (1 + 2)	*T*	*P*/kPa	*x*_1_	*N*_*s*,*p*_^est^	*N*_*s*,*p*_^tot^	%AAD	AAD/K	refs	Figures
naphthalene + phenol	bub.	13.3	0.05–0.56	0	5	0.07795	0.3100	^165^	N/A
naphthalene + ethyl acetate	bub.	98.0	0.01–0.09	0	16	0.4279	1.494	^166^	N/A
phenanthrene + fluoranthene	bub.	101.3	0.00–1.00	0	6	0.7112	4.552	^149^	[Fig fig7]
anthracene + fluoranthene	bub.	101.3	0.00–1.00	0	6	0.7460	4.738	^149^	[Fig fig7]
biphenyl + benzoic acid	bub.	13.3–100.0	0.10–0.90	9	54	1.387	6.759	^167^	N/A
biphenyl + phenylmethyl acetate	bub.	26.7–100.0	0.10–0.90	0	36	0.9740	4.743	^168^	N/A
styrene + benzene	bub.	101.3	0.20–0.98	0	11	0.7105	2.729	^262^	[Fig fig11](d)
styrene + benzene	dew	101.3	0.02–0.83	0	11	0.8198	3.199	^262^	[Fig fig11](d)
styrene + 1,3-butadiene	bub.	98.99–445.3	0.25–1.00	0	17	4.1323	13.70	^263^	N/A
styrene + 1,3-butadiene	dew	98.99–445.3	0.00–0.62	0	17	0.8078	2.590	^263^	N/A
styrene + α-methylstyrene	bub.	20.1–101.3	0.00–1.00	33	33	0.05352	0.2067	^249,264^	[Fig fig11](d)
styrene + α-methylstyrene	dew	20.1–25.0	0.00–1.00	22	22	0.07471	0.2823	^249^	[Fig fig11](d)
2-methyl-1-butene + toluene	bub.	101.3	0.01–0.93	0	13	1.111	3.790	^265^	N/A
2-methyl-1-butene + toluene	dew	101.3	0.09–1.00	0	13	0.4567	1.501	^265^	N/A
isoprene + toluene	bub.	101.3	0.00–1.00	0	13	1.459	5.002	^266^	N/A
isoprene + toluene	dew	101.3	0.00–1.00	0	13	0.5630	1.854	^266^	N/A
styrene + toluene	bub.	101.3	0.10–0.90	0	10	0.1716	0.6907	^262^	[Fig fig11](d)
styrene + toluene	dew	101.3	0.04–0.79	0	10	0.1574	0.6350	^262^	[Fig fig11](d)
styrene + 1,2-dimethylbenzene	bub.	15.0	0.03–0.95	0	19	0.5014	1.793	^248^	[Fig fig11](d)
styrene + 1,2-dimethylbenzene	dew	15.0	0.03–0.95	0	19	0.4668	1.669	^248^	[Fig fig11](d)
styrene + 1,3-dimethylbenzene	bub.	15.0	0.03–0.91	20	20	0.1678	0.5955	^248^	[Fig fig11](d)
styrene + 1,3-dimethylbenzene	dew	15.0	0.03–0.89	20	20	0.1618	0.5741	^248^	[Fig fig11](d)
styrene + 1,4-dimethylbenzene	bub.	15.0	0.05–0.95	0	18	0.3156	1.118	^248^	[Fig fig11](d)
styrene + 1,4-dimethylbenzene	dew	15.0	0.04–0.94	0	18	0.2969	1.052	^248^	[Fig fig11](d)
α-methylstyrene + 1,2-dimethylbenzene	bub.	101.3	0.00–0.95	0	10	0.3474	1.470	^264^	N/A
1-hexene + ethylbenzene	dew	101.3	0.00–1.00	22	22	0.1323	0.4820	^267^	N/A
1-hexene + ethylbenzene	bub.	101.3	0.00–1.00	22	22	0.1014	0.3797	^267^	N/A
1-octene + ethylbenzene	bub.	101.3	0.04–0.95	0	16	0.07338	0.2954	^268^	N/A
1-octene + ethylbenzene	dew	101.3	0.07–0.96	0	16	0.1030	0.4152	^268^	N/A
styrene + ethylbenzene	bub.	20.0	0.00–1.00	0	14	0.1139	0.4113	^269^	[Fig fig11](d)
styrene + ethylbenzene	dew	20.0	0.00–1.00	0	14	0.1039	0.3750	^269^	[Fig fig11](d)
styrene + propylbenzene	bub.	6.7	0.33–0.95	0	6	0.4974	1.694	^270^	[Fig fig11](d)
styrene + propylbenzene	dew	6.7	0.43–0.97	0	6	0.5550	1.889	^270^	[Fig fig11](d)
1-hexene + methanol	bub.	101.3	0.00–1.00	13	13	0.6214	2.008	^271^	N/A
1-hexene + methanol	dew	101.3	0.00–1.00	13	13	0.4168	1.344	^271^	N/A
1-heptene + methanol	bub.	101.3	0.05–1.00	0	12	0.6546	2.185	^272^	N/A
1-heptene + methanol	dew	101.3	0.18–1.00	0	12	0.5075	1.695	^272^	N/A
1-octene + methanol	bub.	101.3	0.00–1.00	0	13	0.4341	1.479	^272^	N/A
1-octene + methanol	dew	101.3	0.00–1.00	0	13	0.4355	1.476	^272^	N/A
isoprene + methanol	bub.	99.3–101.3	0.00–1.00	0	46	0.3931	1.237	^273–276^	N/A
isoprene + methanol	dew	99.3–101.3	0.08–0.84	0	35	0.3188	0.9929	^273–275^	N/A
styrene + propan-1-ol	bub.	101.3	0.02–0.96	0	26	0.3662	1.169	^277^	N/A
styrene + propan-1-ol	dew	101.3	0.02–0.66	0	26	0.1506	0.4724	^277^	N/A
isoprene + butanone	bub.	101.3	0.00–1.00	13	13	2.309	7.541	^278^	N/A
isoprene + pentan-2-one	bub.	101.3	0.00–1.00	0	11	2.657	8.869	^276^	N/A
α-methylstyrene + 4-methyl-3-penten-2-one	bub.	15.5	0.12–0.89	0	7	1.586	5.808	^279^	N/A
α-methylstyrene + 4-methyl-3-penten-2-one	dew	15.5	0.03–0.74	0	7	0.7261	2.627	^279^	N/A
α-methylstyrene + butyl acetate	bub.	13.3–101.3	0.00–1.00	0	33	0.8147	3.186	^250^	N/A
phenol + 1-octene	bub.	13.3	0.03–0.97	9	9	0.2950	1.063	^280^	N/A
phenol + 1-octene	dew	13.3	0.01–0.50	9	9	0.4553	1.605	^280^	N/A
phenol + 1-nonene	bub.	101.3	0.00–1.00	0	14	0.2656	1.162	^281^	N/A
phenol + 1-nonene	dew	101.3	0.00–1.00	0	14	0.1818	0.7799	^281^	N/A
styrene + phenol	bub.	1.3	0.00–1.00	0	11	0.3062	0.9856	^282^	[Fig fig11](d)
styrene + phenol	dew	1.3	0.00–1.00	0	11	0.4978	1.603	^282^	[Fig fig11](d)
α-methylstyrene + phenol	bub.	13.3	0.00–1.00	0	10	0.2503	0.9609	^283^	N/A

*Px* Diagram									
system *s* (1 + 2)	*T*/K	*P*	*x*_1_	*N*_*s*,*p*_^est^	*N*_*s*,*p*_^tot^	%AAD	AAD/kPa	refs	Figures
naphthalene + cyclohexane	372–480	bub.	0.11–0.72	30	30	2.017	7.972	^169^	N/A
naphthalene + methanol	521–580	bub.	0.05–0.89	0	22	6.943	265.0	^170^	[Fig fig11](a)
naphthalene + methanol	521–580	dew	0.03–0.28	0	22	33.24	1690	^170^	[Fig fig11](a)
naphthalene + acetone	298	bub.	0.00–0.15	3	3	0.7004	0.1983	^171^	[Fig fig11](b)
naphthalene + butanone	298	bub.	0.13–0.47	5	18	8.600	3.433	^172^	[Fig fig11](b)
naphthalene + butanone	298	dew	0.00–0.01	5	18	3.749	2.030	^172^	[Fig fig11](b)
naphthalene + phenol	363	bub.	0.02–0.08	0	15	3.392	0.06998	^172^	N/A
naphthalene + phenol	363	dew	0.03–0.10	0	15	3.370	70.18	^172^	N/A
phenanthrene + methanol	426–538	bub.	0.936–0.942	24	24	25.43	132.2	^173^	N/A
phenanthrene + ethanol	427–538	bub.	0.924–0.930	24	24	5.493	25.62	^173^	N/A
phenanthrene + propan-1-ol	427–539	bub.	0.910–0.914	24	24	4.405	14.25	^173^	N/A
phenanthrene + butan-1-ol	446–538	bub.	0.88–0.89	20	20	1.254	3.682	^173^	N/A
phenanthrene + pentan-1-ol	464–542	bub.	0.901–0.904	17	17	26.21	51.37	^173^	N/A
phenanthrene + acetone	298	bub.	0.00–0.10	3	3	0.5080	0.1479	^171^	N/A
anthracene + acetone	293–313	bub.	0.003–0.005	3	3	10.80	3.481	^174^	N/A
1,3-butadiene + benzene	298–413	bub.	0.00–1.00	50	50	4.609	0.04022	^284,285^	N/A
1,3-butadiene + benzene	298–413	dew	0.00–1.00	50	50	15.17	0.1478	^284,285^	N/A
isoprene + benzene	278–298	bub.	0.00–1.00	55	55	2.260	0.5199	^286^	N/A
1,3-butadiene + toluene	303–413	bub.	0.00–1.00	0	47	14.34	119.2	^284^	N/A
1,3-butadiene + toluene	303–413	dew	0.00–1.00	0	47	18.07	173.2	^284^	N/A
α-methylstyrene + toluene	293–313	bub.	0.00–1.00	0	48	19.34	0.5692	^261^	N/A
α-methylstyrene + acetone	296–372	bub.	0.00–1.00	0	45	10.58	4.092	^287^	N/A
1-hexene + butanone	333	bub.	0.09–0.91	11	11	2.526	1.845	^288^	N/A
1-hexene + butanone	333	dew	0.27–0.89	11	11	2.563	1.950	^288^	N/A
1-hexene + ethyl acetate	333	bub.	0.00–1.00	24	24	0.5428	0.4661	^289^	N/A
1-hexene + ethyl acetate	333	dew	0.00–1.00	0	24	0.5755	0.4826	^289^	N/A
1-octene + ethyl acetate	348	bub.	0.00–1.00	0	25	1.181	0.6418	^289^	N/A
1-octene + ethyl acetate	348	dew	0.00–1.00	0	25	0.9386	0.6521	^289^	N/A
phenol + 1-nonene	393–413	bub.	0.00–1.00	0	36	3.357	1.441	^290^	N/A
phenol + 1-undecene	428–443	bub.	0.00–1.00	0	34	1.867	1.110	^290^	N/A
styrene + phenol	333–373	bub.	0.00–1.00	0	30	8.210	0.7541	^291^	N/A

Density of Binary Mixture									
system *s* (1 + 2)	*T*/K	*P*/kPa	*x*_1_	*N*_*s*,*p*_^est^	*N*_*s*,*p*_^tot^	%AAD	AAD/(kg/m^3^)	refs	Figures
naphthalene + acetone	298	101.3	0.23	1	1	0.0006558	0.5756	^175^	N/A
phenanthrene + ethyl acetate	288–343	101.3	0.03–0.09	0	18	0.3085	2.749	^110^	N/A
anthracene + acetone	298	101.3	0.004	0	1	0.006758	5.376	^176^	N/A
biphenyl + ethyl acetate	298	101.3	0.01	0	5	0.1274	1.159	^110^	N/A
2-methyl-2-butene + benzene	292	101.3	0.00–0.46	0	5	2.574	21.61	^292^	N/A
styrene + benzene	298	101.3	0.00–1.00	0	30	1.673	14.87	^293,294^	N/A
styrene + toluene	298	101.3	0.00–1.00	0	23	1.088	9.673	^295^	N/A
styrene + 1,2-dimethylbenzene	298–313	101.3	0.00–1.00	0	67	1.677	14.83	^296,297^	N/A
styrene + 1,3-dimethylbenzene	298–313	101.3	0.00–1.00	0	67	0.8127	7.187	^297,298^	N/A
styrene + 1,4-dimethylbenzene	298–313	101.3	0.00–1.00	0	67	0.8155	7.193	^297,299^	N/A
1-hexene + ethylbenzene	298	101.3	0.00–1.00	0	21	0.6072	4.621	^300^	N/A
styrene + ethylbenzene	298	101.3	0.00–1.00	0	46	0.7334	6.532	^301,302^	N/A
styrene + heptane	298	101.3	0.00–1.00	0	20	0.6820	5.657	^303,304^	N/A
styrene + octane	293–363	101.3	0.00–1.00	0	121	0.8111	6.652	^257,304^	N/A
styrene + cyclohexane	298	101.3	0.00–1.00	0	23	1.188	10.12	^305^	N/A
isoprene + methanol	293	101.3	0.05–1.00	0	10	1.699	11.86	^306^	N/A
styrene + methanol	298	101.3	0.00–1.00	0	11	0.8523	7.535	^307^	N/A
styrene + ethanol	298	101.3	0.00–1.00	0	11	1.047	8.997	^307^	N/A
styrene + propan-1-ol	298–308	101.3	0.00–1.00	0	33	1.496	12.84	^307^	N/A
styrene + butan-1-ol	298–308	101.3	0.00–1.00	0	32	0.9462	8.111	^307^	N/A
styrene + hexan-1-ol	298	101.3	0.00–1.00	0	11	0.8963	7.738	^307^	N/A
styrene + acetone	298–313	101.3	0.00–1.00	0	44	0.9197	7.990	^308^	N/A
styrene + ethyl acetate	298	101.3	0.00–1.00	0	30	1.322	11.93	^294,309^	N/A

Excess Molar Enthalpy									
system *s* (1 + 2)	*T*/K	*P*/kPa	*x*_1_	*N*_*s*,*p*_^est^	*N*_*s*,*p*_^tot^	%AAD	AAD/(J/mol)	refs	Figures
1-hexene + methanol	298–328	101.3–500.0	0.05–0.98	59	59	13.89	75.08	^310,311^	N/A
1-heptene + methanol	298	101.3	0.08–0.91	0	11	33.82	162.0	^310^	N/A
1-octene + methanol	298–328	101.3–500.0	0.02–0.95	0	60	29.26	168.3	^310,311^	N/A
1-hexene + butanone	298–328	500.0	0.04–0.95	0	49	21.61	114.9	^312^	N/A
1-hexene + ethyl acetate	298–343	600.0	0.03–0.95	9	37	11.22	42.11	^313^	N/A
1-octene + ethyl acetate	298–343	600.0	0.03–0.94	0	55	8.961	50.00	^313^	N/A

a *N*_*s*,*p*_^est^ is the number of experimental data used in parameter estimation, and *N*_*s*,*p*_^tot^ is the number of experimental data used to calculate AAD_*s*_ [*p*], for system *s* and property *p.*

Examples of SLE predictions for mixtures of aromatic hydrocarbons are shown in Figure 5. Mixtures of naphthalene + benzene derivatives (Figure 5(a),(b)), as well as naphthalene +3-ring PAHs (Figure 5(c)), exhibit eutectic behavior. The SAFT-γ Mie predictions for these systems are in very good agreement with the experimental data. Chrysene + pyrene also constitutes a eutectic system, while chrysene and benz­[*a*]­anthracene are miscible in the solid phase (Figure 5(d)). In particular, chrysene and benz­[*a*]­anthracene are modeled with the same SAFT-γ Mie groups (six aC and 12 aCH groups) but differ in their melting point and enthalpy of fusion (cf. Table 2). As a consequence, the SLE of chrysene + benz­[*a*]­anthracene predicted with SAFT-γ Mie corresponds to the ideal SLE of two compounds miscible in the solid phase, which is in excellent agreement with the experimental data, both for the liquidus and the solidus. These SLE calculations constitute a new application and validation of the SAFT-γ Mie group interactions characterized recently^23^ for the aC–aC, aC–aCH, aC–aCCH_3_, aC–aCCH_2_, aC–CH_3_, and aC–CH_2_ interactions.

**Figure 5:**
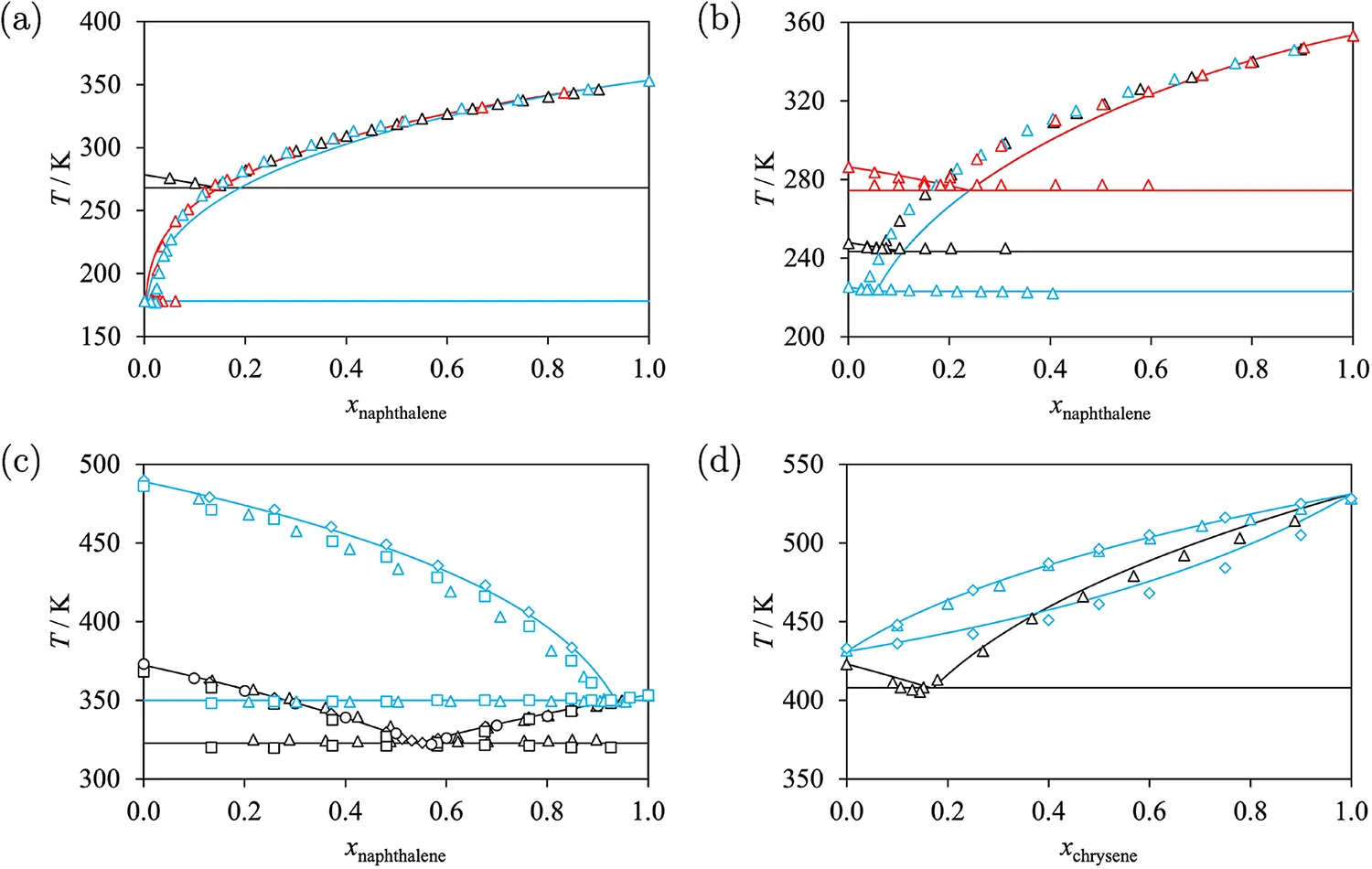
Isobaric solid–liquid equilibria at atmospheric pressure of mixtures of aromatic hydrocarbons. The curves represent the predictions with SAFT-γ Mie. Experimental data (not used in the estimation of group-interaction parameters) are represented with open symbols. (a) Naphthalene + benzene derivatives: benzene^99^ (black), toluene^100^ (light blue), and ethylbenzene^101^ (red). (b) Naphthalene + dimethylbenzenes: 1,2-dimethylbenzene^101^ (black), 1,3-dimethylbenzene^100^ (light blue), and 1,4-dimethylbenzene^101^ (red). (c) Naphthalene + PAHs with three fused rings: phenanthrene^15,86–88^ (black) and anthracene^87,88,105^ (light blue). (d) Chrysene + pyrene^87^ (black) and chrysene + benz­[*a*]­anthracene^16^ (light blue). The thermodynamic conditions and accuracy of the theoretical description are detailed in Table 5.

Models for phenyls contain aC groups that can form bridges between nonfused aromatic cycles. Biphenyl is modeled with ten aCH and two aC groups that create an aC–aC bridge (cf. Figures 1 and 3(b)). Terphenyls are modeled with 14 aCH and four aC groups that create two aC–aC bridges. Ortho (*o*-), meta (*m*-), and para (*p*-) terphenyls exhibit different melting points and enthalpies of fusion, and their SLE in solvents can be distinguished. It should be noted that the aC group was not parametrized with data involving systems where there is a bridging carbon between phenyl moieties. As such, this is an unexplored extension of the group’s applicability. In Figure 6, we show the SAFT-γ Mie prediction for the vapor pressure (Figure 6(a)) and the saturation density (Figure 6(b)) of pure biphenyl, and the isobaric SLE of phenyls + benzene (Figure 6(c)) and phenyls + naphthalene (Figure 6(d)). The SLE curves correspond to eutectic systems, and excellent quantitative agreement is found with the experimental data. In particular, deviations for the SLE composition exhibit AAD = 0.0186 for biphenyl + benzene and AAD = 0.0160 for biphenyl + naphthalene. These findings confirm that the aC group can be used to model unfused aromatic compounds, including phenyls, and to predict their thermodynamic properties.

**Figure 6:**
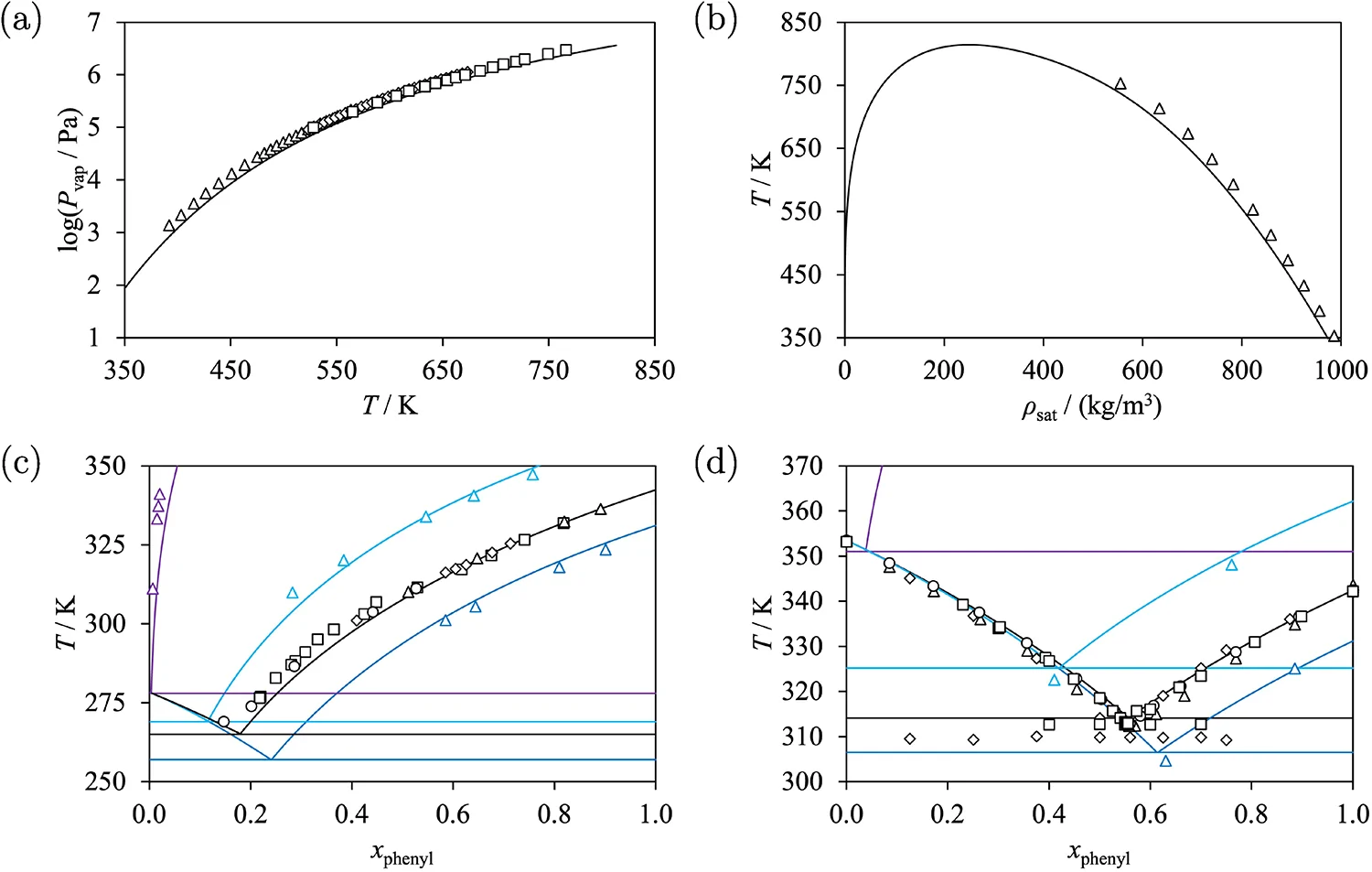
Selected thermodynamic properties of pure phenyl and phenyl mixtures. The curves represent the predictions with SAFT-γ Mie for biphenyl (black) and terphenyls: *o*-terphenyl (dark blue), *m*-terphenyl (light blue), and *p*-terphenyl (purple). Experimental data (not used in the estimation of group-interaction parameters) are represented with open symbols. (a) Vapor pressure of pure biphenyl.^145–147^ (b) Saturation temperature–density diagram of pure biphenyl.^148^ (c) Isobaric solid–liquid equilibrium at atmospheric pressure of phenyls + benzene, with experimental data for biphenyl^130–132,134^ and terphenyls.^134^ (d) Isobaric solid–liquid equilibrium at atmospheric pressure of phenyls + naphthalene, with experimental data for biphenyl,^87,90–92^ *o*-terphenyl,^93^ and *m*-terphenyl.^93^ The thermodynamic conditions and accuracy of the theoretical description are detailed in Tables 5 and 6.

We also use combinations of the aCH and aC groups to model PAHs that contain five-membered rings. Fluoranthene can be modeled with six aC groups and ten aCH groups to form three six-membered aromatic cycles and one five-membered aromatic cycle (cf. Figure 1). The isobaric phase diagrams at atmospheric pressure of fluoranthene + phenanthrene and fluoranthene + anthracene can be seen in Figure 7. As phenanthrene and anthracene are modeled with the same SAFT-γ Mie groups, the predicted VLE for the fluoranthene + phenanthrene mixture is identical to the predicted VLE for fluoranthene + anthracene, while a small difference can be seen in the experimental VLE data for these two systems.^149^ However, the agreement between SAFT calculations and experiments is very good for both mixtures, with deviations of 0.7112% for fluoranthene + phenanthrene and 0.7460% for fluoranthene + anthracene, which makes the use of the group-contribution approach to predict the thermophysical properties of these fluids acceptable. Phenanthrene and anthracene have, however, different fusion properties, such that the predicted SLE curves for fluoranthene + phenanthrene and fluoranthene + anthracene are significantly different. The predicted SLE results are consistent with the experimental data for both mixtures.

**Figure 7:**
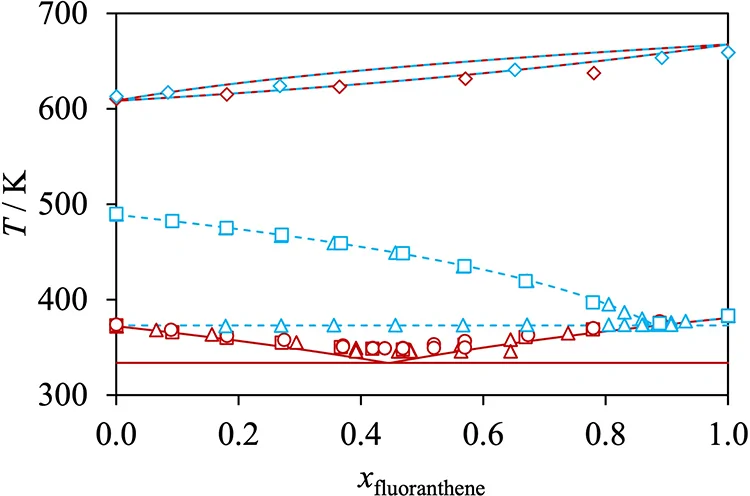
Isobaric phase diagram at atmospheric pressure of fluoranthene + phenanthrene (dark red) and fluoranthene + anthracene (light blue). The curves represent the predictions with SAFT-γ Mie. The red curves correspond to the prediction for fluoranthene + phananthrene, and the blue curves correspond to the prediction for fluoranthene + anthracene. Experimental data (not used in the estimation of group-interaction parameters) are represented with open symbols for the VLE^149^ and SLE.^87,94,97^ The thermodynamic conditions and accuracy of the theoretical description are detailed in Tables 5 and 6.

### 3.2 Application of New SAFT-γ Mie Group-Interaction Parameters for Aromatic Compounds in Various Organic Solvents

The group-interaction parameters characterized in our current work are used to model partially saturated PAHs, binary mixtures of PAHs + various aliphatic compounds, binary mixtures of PAHs + functionalized aromatic compounds, and systems that contain styrene derivatives. Extensive experimental data are available for the thermophysical properties of the tested pure PAHs and functionalized polycyclic aromatic compounds, including the vapor pressure,^150–158^ density of pure fluids,^148,150,159–161^ and enthalpy of vaporization.^148,150,157,160,162–164^ Moreover, data are also available for binary mixtures of PAHs + alkanes and PAHs + functionalized compounds, including isobaric VLE,^149,165–168^ isothermal VLE,^169–174^ density of mixtures,^110,175,176^ and isobaric SLE.^55,56,88,99,111,112^,​^114,121–123,126,130^,​^132,139–143,172^,​^176–196^​^197,198,199,200,201,202,203,204,205,206,207,208,209,210,211,212,213,214,215,216,217,218,219,220,221,222,223,224,225,226,227,228,229,230,231,232,233,234,235,236,237,238,239,240,241^ For systems that contain nonaromatic unsaturated bonds (e.g., styrene and alkenes), experimental data for the vapor pressure,^242–254^ density of pure fluids,^252,255–261^ isobaric VLE,^248–250,262–283^ isothermal VLE,^261,284–291^ density of mixtures,^257,292–309^ and excess molar enthalpy^310–313^ are used.

We use the SAFT-γ Mie approach to study the properties and phase behavior of partially saturated PAHs, e.g., fluorene (cf. Figures 1 and 3(c)), and acenaphthene (cf. Figure 1). The characterization of the aC–cCH_2_ group interactions is required to model partially saturated PAHs, as well as mixtures of PAHs + cycloalkanes. In Figure 8, we give examples of results obtained after characterization. The vapor pressure (Figure 8(a)), saturated density (Figure 8(b)), and enthalpy of vaporization (Figure 8(c)) are predicted for pure fluorene and acenaphthene with very good overall agreement with the experimental data. We also obtain excellent predictions for the solubility mole fraction of naphthalene + cycloalkanes (Figure 8(d)), with AAD = 0.0144 for naphthalene + cyclopentane, and AAD = 0.0172 for naphthalene + cyclohexane. This confirms the versatility of the group-contribution approach and the applicability of the aC–cCH_2_ interactions.

**Figure 8:**
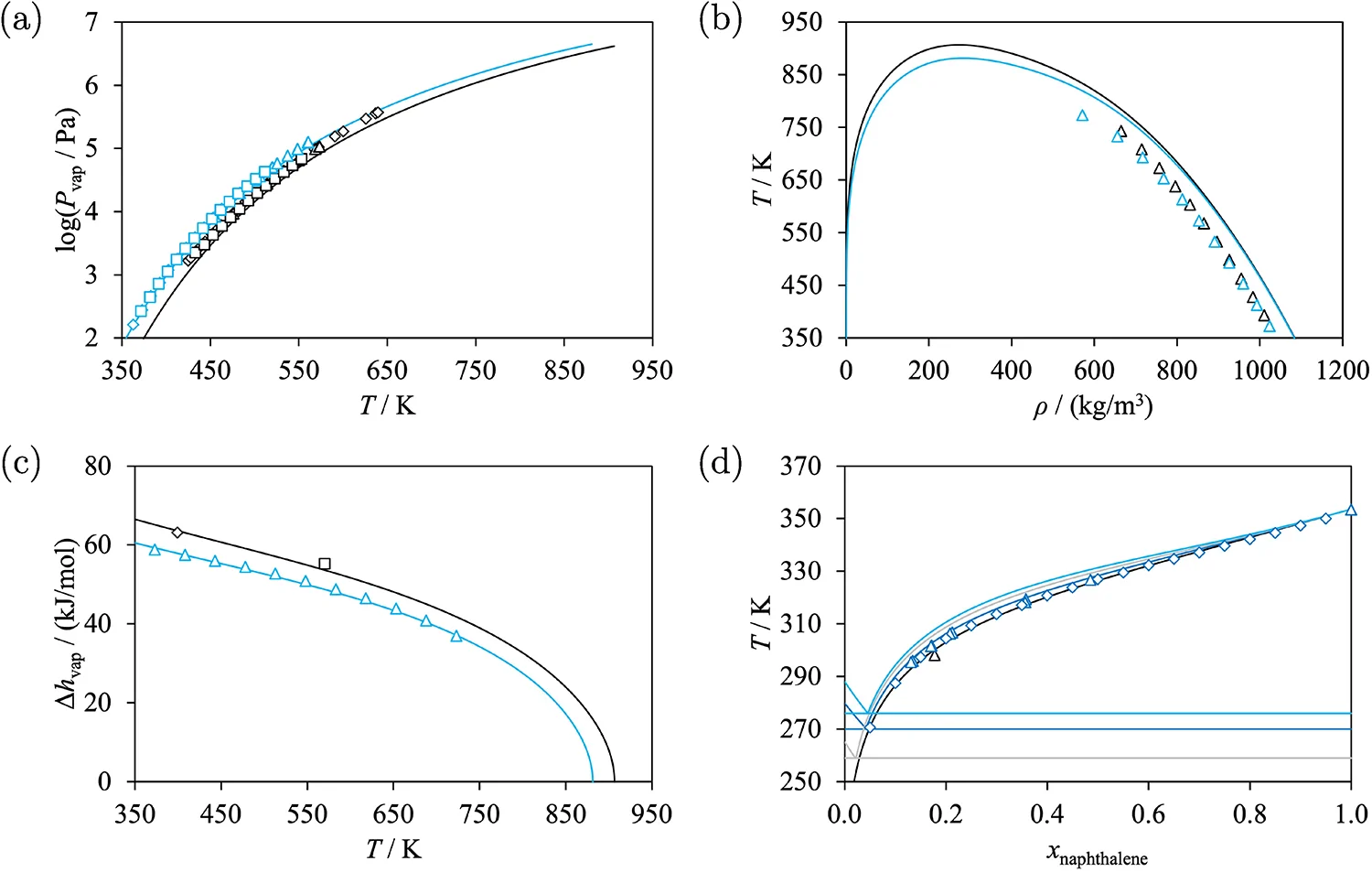
Selected thermodynamic properties of systems containing the aC and cCH_2_ groups. The curves represent the calculations with SAFT-γ Mie. Experimental data (not used in the estimation of group-interaction parameters) are represented with open symbols. (a) Vapor pressure of fluorene (black) and acenaphthene (light blue), with experimental data for fluorene^151–153^ and acenaphthene.^151,154,155^ (b) Saturation temperature–density diagram of fluorene^148^ (black) and acenaphthene^148^ (light blue). (c) Vaporization enthalpy of fluorene^148^ (black) and acenaphthene^74,164^ (light blue). (d) Isobaric solid–liquid equilibrium at atmospheric pressure of naphthalene + cycloalkanes: cyclopentane^188^ (black), cyclohexane^99,178^ (dark blue), cycloheptane (gray), and cyclooctane (light blue). The thermodynamic conditions and accuracy of the theoretical description are detailed in Tables 5 and 6.

For the modeling of other PAHs + organic solvents, here we also characterize the aC–CH, aC–cCH, aC–CH_3_OH, aC–CH_2_OH, aC–CHOH, aC–cCHOH, aC–CH_3_COCH_3_, aC–CH_3_CO, aC–COO, aC–aCOH, and aC–aCCOOH interactions. New group-interaction parameters are developed for unsaturated (nonaromatic) compounds that contain the CH_2_=, CH=, or C= groups, and we characterize the CH_2_=–aC, CH=–aC, C=–aC, CH_2_=–aCH, CH=–aCH, C=–aCH, CH_2_=–aCCH_3_, CH=–aCCH_3_, C=–aCCH_3_, CH_2_=–aCCH_2_, CH=–aCCH_2_, CH_2_=–CH_3_OH, CH=–CH_3_OH, C=–CH_3_OH, CH_2_=–CH_3_CO, CH=–CH_3_CO, C=–CH_3_CO, CH_2_=–COO, CH_2_=–aCOH, CH=–aCOH, and C=–aCOH interactions. These interactions are needed to model compounds that contain the aC group together with the C=, CH=, or CH_2_= groups, e.g., styrene and α-methylstyrene.

In Figure 9, we show SLE curves at atmospheric pressure for naphthalene + linear alkanes (Figure 9(a)), primary alcohols (Figure 9(b)), secondary alcohols (Figure 9(c)), and secondary ketones (Figure 9(d)). The SLE of these mixtures are found to correspond to eutectic systems. Only one experimental eutectic point is shown in Figure 9 for naphthalene + *n*-eicosane. This is because most of the selected solvents have low melting points, out of the temperature range considered in the experiments. The SAFT-γ Mie calculations are in very good overall agreement with the experimental data, and the AADs for the solubility mole fraction (detailed in Table 5) are found to be small (e.g., AADs for naphthalene + solvents are 0.0334 with heptane, 0.0476 with methanol, 0.0624 with ethanol, 0.0333 with propan-2-ol, 0.0563 with acetone, and 0.0511 with butanone). For *T* > 270 K, eutectic points are found for naphthalene + *n*-eicosane (in good agreement with the experimental data) and for naphthalene + decan-1-ol (for this mixture, we cannot evaluate the quality of the prediction due to a lack of experimental data). For the other mixtures shown in Figure 9, the SAFT-γ Mie curves follow the expected behavior for a given family. For instance, the solubility of naphthalene increases when considering longer primary or secondary alcohols at a given temperature above the melting points of the solvents. The results confirm the validity of the new parameters (Table 4) that characterize the aC–CH_3_OH, aC–CH_2_OH, aC–CHOH, aC–CH_3_COCH_3_, and aC–CH_3_CO interactions, as well as the parameters recently characterized for the aC–CH_3_ and aC–CH_2_ group interactions^23^ used in the four solvent families shown in Figure 9.

**Figure 9:**
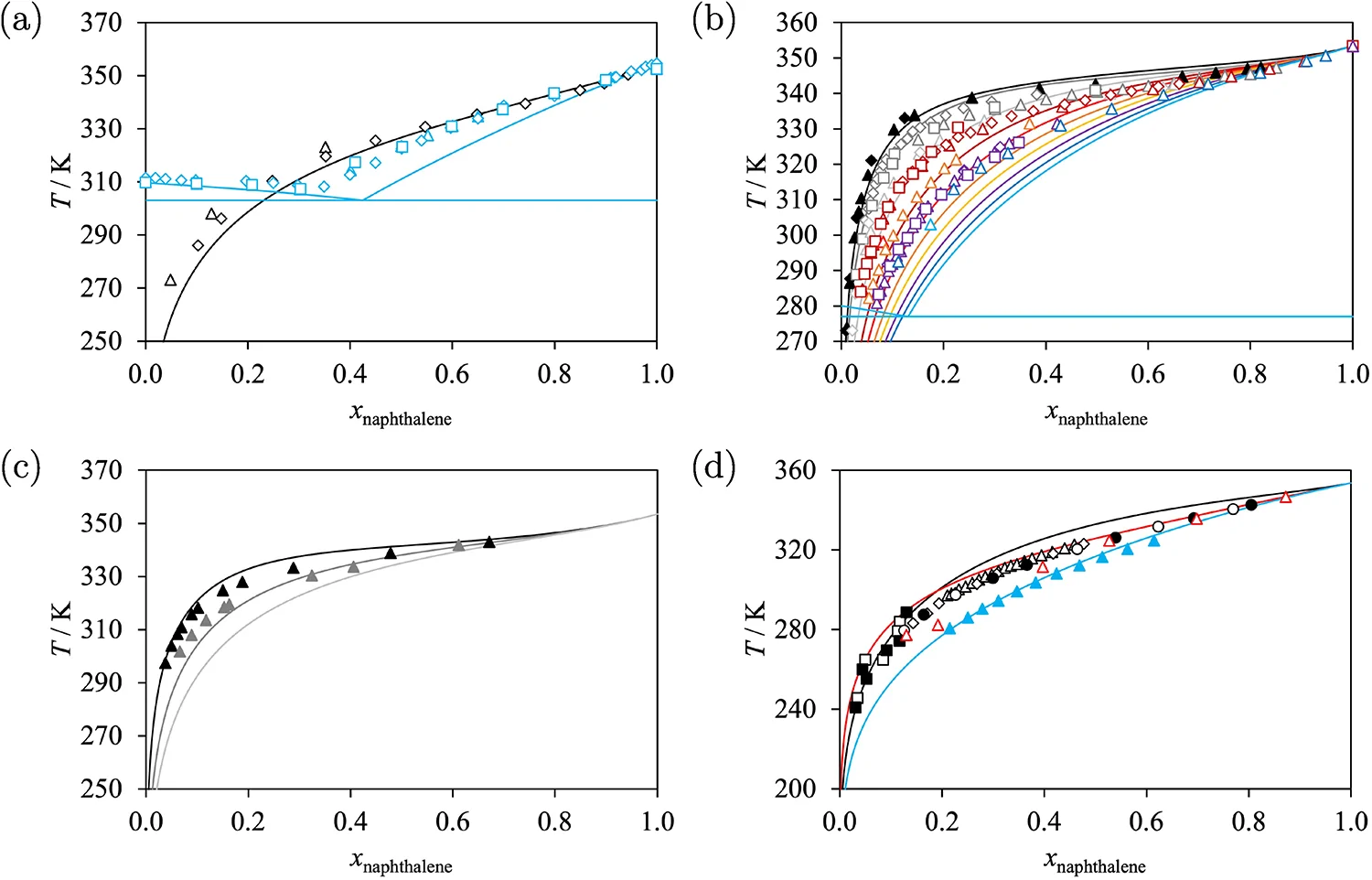
Isobaric solid–liquid equilibrium at atmospheric pressure of naphthalene + solvents. The curves represent calculations with SAFT-γ Mie. Experimental data used in the estimation of group-interaction parameters are represented with filled symbols and those not used with open symbols. (a) Naphthalene + alkanes: *n*-heptane^172,179^ (black) and *n*-eicosane^56,186,187^ (light blue). (b) Naphthalene +1-alcohols: methanol^114,177^ (black), ethanol^99,189,190^ (dark gray), propan-1-ol^114,190^ (light gray), butan-1-ol^177,178,189^ (dark red), pentan-1-ol^191^ (light red), hexan-1-ol^178^ (orange), heptan-1-ol (yellow), octan-1-ol^178,183,192^ (purple), nonan-1-ol^193^ (dark blue), and decan-1-ol^182^ (light blue). (c) Naphthalene +2-alcohols: propan-2-ol^190^ (black), butan-2-ol^190^ (dark gray), and pentan-2-ol (light gray). (d) Naphthalene +2-ketones: acetone^177,189,196,197^ (black), butan-2-one^172^ (red), and octan-2-one^183^ (light blue). The thermodynamic conditions and accuracy of the theoretical description are detailed in Table 5.

It is worth noting that the overall accuracy between the SAFT-γ Mie calculations and the experiments is comparable with other approaches like COSMO or UNIFAC. For example, for naphthalene + methanol, excellent agreement is obtained with SAFT-γ Mie, UNIFAC­(Do), and COSMO-SAC-Phi,^61^ while worse agreement is obtained with the standard COSMO-SAC (cf. Figure 6a of Camargo et al.^61^). For naphthalene + ethanol, better prediction is obtained with SAFT-γ Mie than with the COSMO and UNIFAC approaches (cf. Figure 5d of Camargo et al.^61^). However, for naphthalene + octan-1-ol, deviations seem larger with SAFT-γ Mie than with other approaches (cf. Figure 6b of Camargo et al.^61^).

In Figure 10, we give examples of SLE for PAHs + esters (Figure 10(a)), branched compounds (Figure 10(b)), phenol (Figure 10(c)), and benzoic acid (Figure 10(d)), which all correspond to eutectic systems. Experimental data of naphthalene + ethyl acetate are used to characterize the aC–COO interactions, and the parameters obtained are used to predict the SLE of naphthalene + methyl hexadecanoate and methyl octadecanoate. Our calculations for these two mixtures are in good agreement with the experimental data, including for the eutectic points. Mixtures of naphthalene + branched compounds are tested with isobutyl acetate (composed of three CH_3_, one CH_2_, one CH, and one COO groups) and 2-methyl-1-propanol (composed of two CH_3_, one CH, and one CH_2_OH groups). Only nine experimental points of solubility are available for naphthalene + 2-methyl-1-propanol^190^ (four of which are used to characterize the aC–CH group interaction); the corresponding mole fraction AAD of the SAFT-γ Mie calculations is 0.0473. The parameters are used to predict the solubility of naphthalene + isobutyl acetate, and excellent agreement is obtained, with AAD = 0.00315. Solubility data of naphthalene + phenol are used to characterize the aC–aCOH interactions. The corresponding SAFT-γ Mie calculations are in excellent agreement with experiment (AAD = 0.0506), as well as the predictions for phenanthrene + phenol (AAD = 0.0527) and anthracene + phenol (AAD = 0.00550). Similarly, the SLE of naphthalene + benzoic acid is accurately determined (AAD = 0.0150).

**Figure 10:**
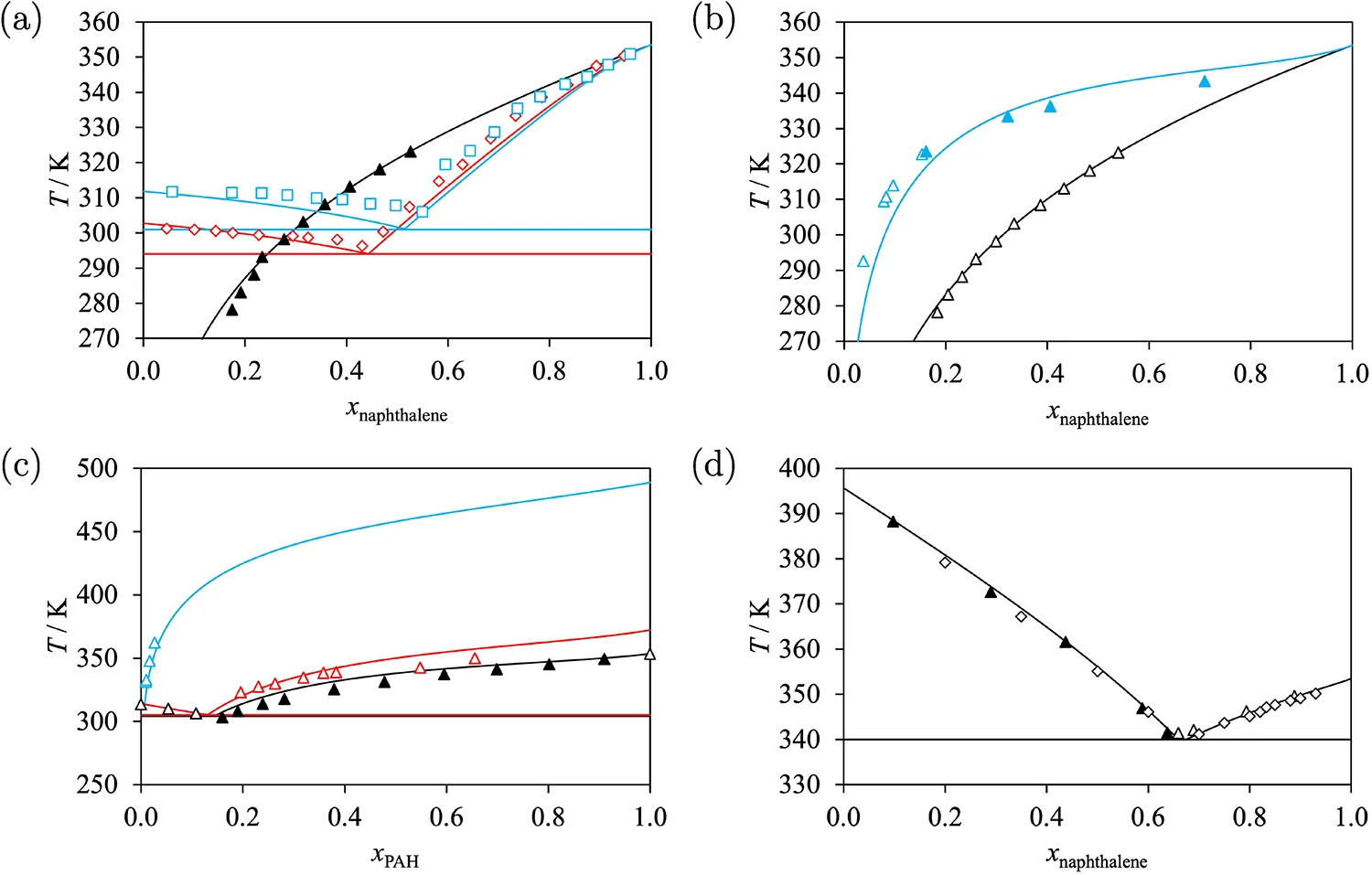
Isobaric solid–liquid equilibrium at atmospheric pressure of PAHs + functionalized compounds. The curves represent calculations with SAFT-γ Mie. Experimental data used in the estimation of group-interaction parameters are represented with filled symbols and those not used with open symbols. (a) Naphthalene + esters: ethyl acetate^196^ (black), methyl hexadecanoate^198^ (red), and methyl octadecanoate^198^ (light blue). (b) Naphthalene + branched compounds: isobutyl acetate^196^ (black) and 2-methyl-1-propanol^190^ (light blue). (c) Naphthalene^199^ (black), phenanthrene^172^ (red), and anthracene^172^ (light blue) + phenol. (d) Naphthalene + benzoic acid.^204,205^ The thermodynamic conditions and accuracy of the theoretical description are detailed in Table 5.

The newly characterized parameters can also be used to determine the VLE properties of pure fluids (e.g., vapor pressure) and mixtures (e.g., bubble and dew temperature or pressure). Examples of VLE calculations and experimental data are shown in Figure 11. Isothermal VLE of naphthalene + methanol (Figure 11(a)) are provided at different temperatures, including for supercritical methanol. We also give examples of isothermal VLE of naphthalene + acetone and naphthalene + butan-2-one (Figure 11(b)). The vapor pressures of pure 1-naphthalenol and 2-naphthalenol are also provided (Figure 11(c)). These two compounds are modeled with the same SAFT-γ Mie groups, such that their calculated vapor pressures are identical. Additionally, bubble and dew temperature of styrene + aromatic compound mixtures are shown in a parity plot (Figure 11(d)). For all the systems and properties shown in Figure 11, the fluid-phase transitions are correctly predicted and excellent overall agreement between the SAFT-γ Mie calculations and experimental data is found. AADs are detailed in Table 6 for the systems shown in Figure 11 and for many additional systems. In particular, styrene and α-methylstyrene are used to confirm the versatility of the characterized parameters, as the modeling of these compounds (which are not PAHs) involves the aC group.

**Figure 11:**
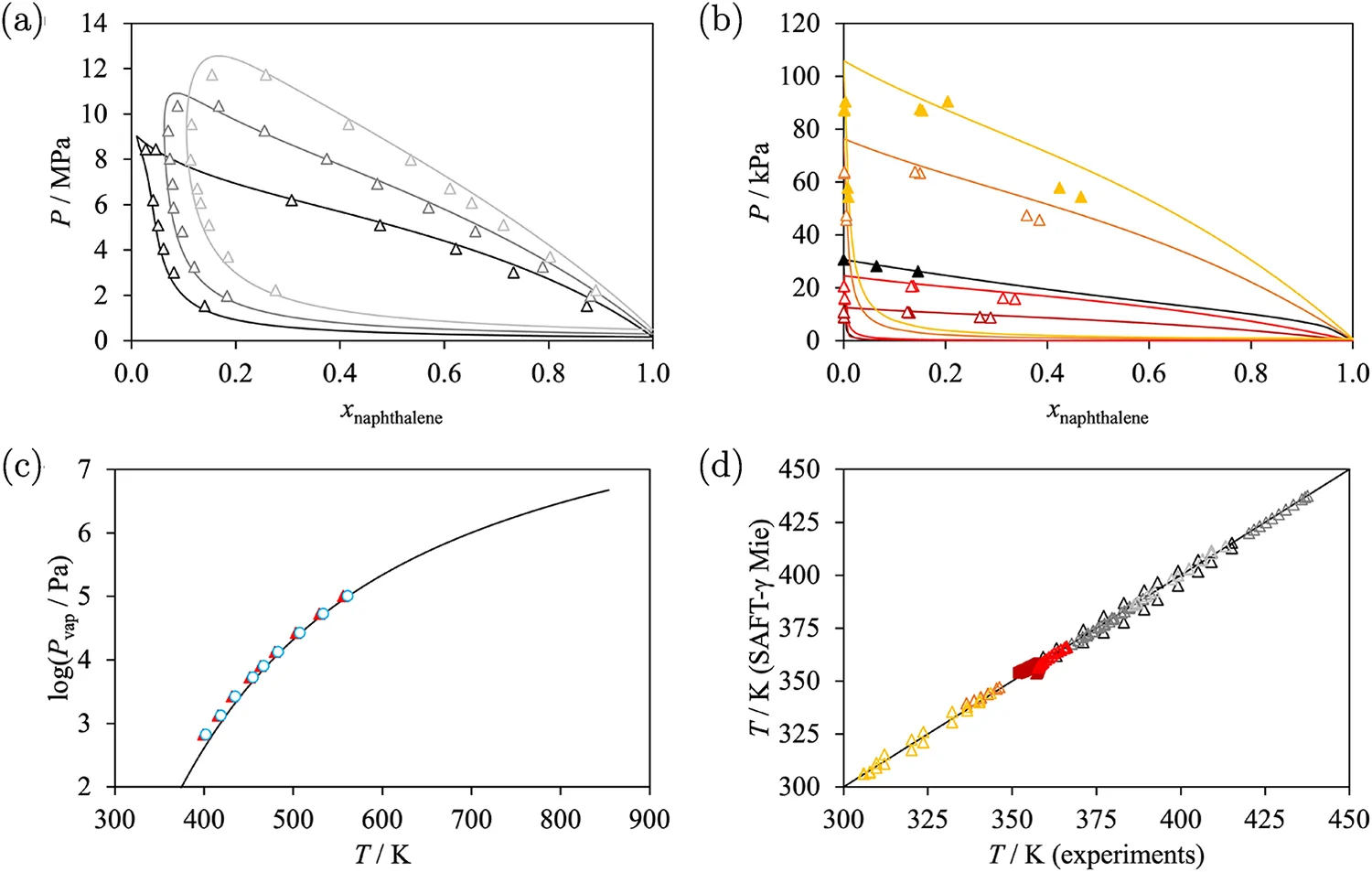
Selected vapor–liquid equilibria of aromatic compounds that contain the aC group. The curves represent the calculations with SAFT-γ Mie. Experimental data used in the estimation of group-interaction parameters are represented with filled symbols and those not used with open symbols. (a) Isothermal vapor–liquid equilibria of naphthalene + methanol^170^ at 521.25 K (black), 549.55 K (dark gray), and 579.75 K (light gray). (b) Isothermal vapor–liquid equilibria of naphthalene + acetone^171^ at 298.15 K (black), and naphthalene + butan-2-one^172^ at 298.15 K (dark red), 313.15 K (red), 343.15 K (orange), and 353.15 K (yellow). (c) Vapor pressure of pure 1-naphthalenol^158^ (red) and pure 2-naphthalenol^158^ (blue), with SAFT-γ Mie description (black). (d) Parity plot for the isobaric vapor–liquid equilibria of styrene + aromatic compounds:^248,249,262,264,269,270,282^ benzene (black), α-methylstyrene (dark gray), toluene (light gray), dimethylbenzene (dark red), ethylbenzene (red), propylbenzene (orange), and phenol (yellow). The thermodynamic conditions and accuracy of the theoretical description are detailed in Tables 5 and 6.

### 3.3 Overall Agreement and AAD

We summarize the accuracy of the SAFT-γ Mie calculations for the solubility of PAHs + organic solvents in the heatmap shown in Figure 12. This graphical representation is convenient to present together the AADs and the number of experimental points found for a given mixture.^314–316^ Four common PAHs are considered: naphthalene, phenanthrene, anthracene, and biphenyl. We study mixtures of these PAHs with several aromatic hydrocarbons (fused PAHs, terphenyls, and monocyclic aromatic hydrocarbons), as well as with other organic solvents representative of chemical families: *n*-heptane for linear alkanes, cyclohexane for cyclic alkanes, methanol and ethanol for primary alcohols, propan-2-ol for secondary alcohols, acetone and butanone for ketones, ethyl acetate for esters, 2-methyl-1-propanol for branched compounds, and phenol and benzoic acid for functionalized aromatic compounds. This choice of organic solvents is relevant for testing the different SAFT-γ Mie group interactions. The circled area for a system *s* in the heatmap is proportional to the number of experimental data points *N*_*s*_^*D*,total^ used to calculate the AADs (the maximum is 87 points for *s* = phenanthrene + anthracene), and a given value of AAD is represented with a color. The minimum is AAD = 0.000803 for anthracene + methanol, and the maximum is AAD = 0.220 for phenanthrene + *n*-heptane. We note that there is no correlation between *N*_*s*_^*D*,total^ and AADs (the correlation coefficient is 0.0945 only), cf. Figure 12. We have not considered peritectic and dystectic systems (e.g., naphthalene +1-methylnaphthalene^87,105^ and phenanthrene + 2-methylnaphthalene^15,116^ ) as their corresponding SLE are described with different equations^79^ and go beyond the scope of our current work.

**Figure 12:**
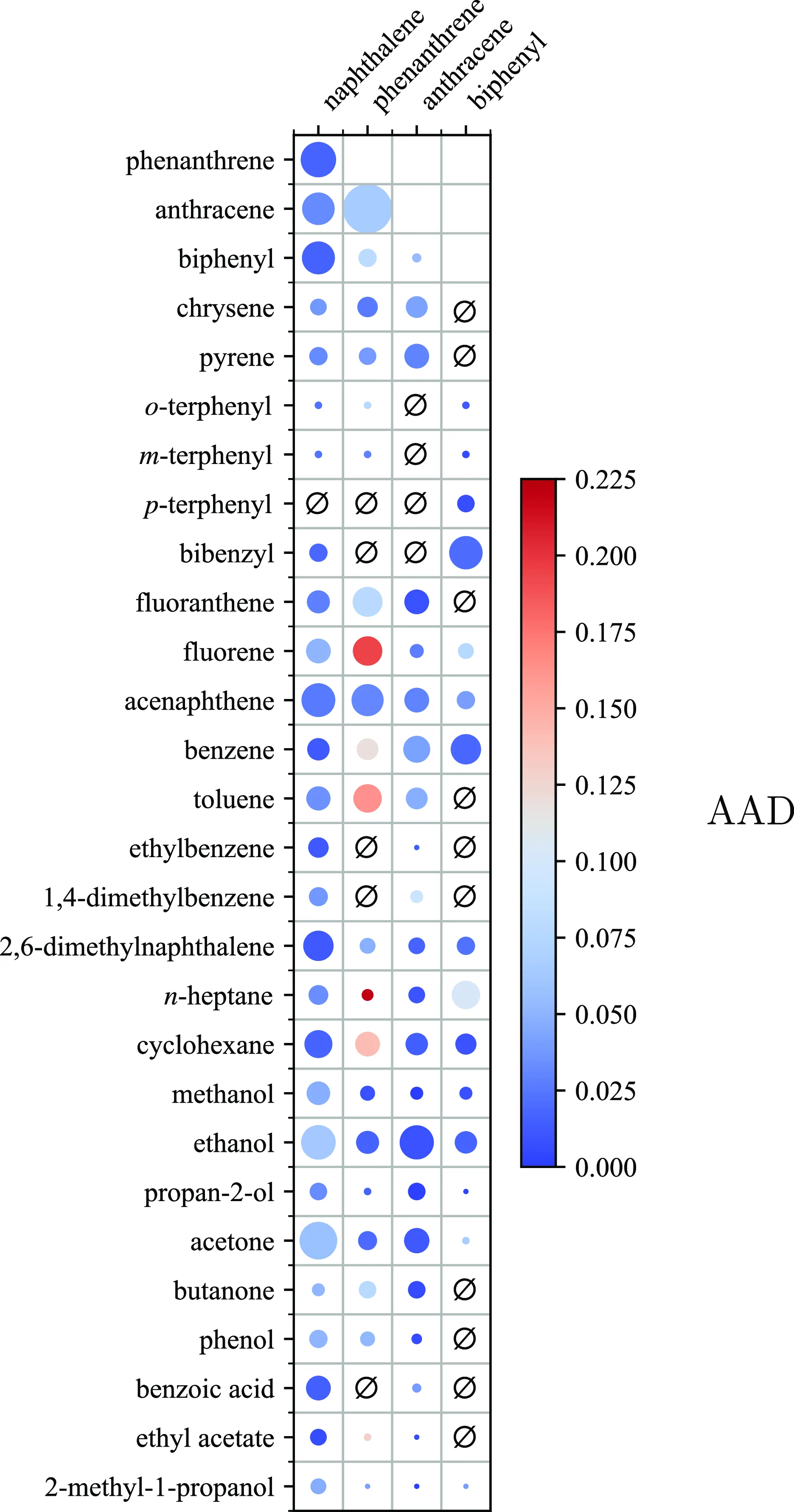
Heatmap of the accuracy of SAFT-γ Mie calculations in terms of mole fraction AAD solubility *x* ^sat^ for PAHs (naphthalene, phenanthrene, anthracene, and biphenyl, indicated by the columns) + organic solvents (given by the rows). AADs are represented with a color scale from blue to red. The circled area is proportional to the number of experimental data points considered. The absence of experimental data in the literature is denoted by the empty set symbol (Ø). The top-right cells are empty by symmetry as phenanthrene, anthracene, and biphenyl are present both in columns and rows.

We have not found experimental SLE data (using the DETHERM database) for: naphthalene + *p*-terphenyl; phenanthrene + *p*-terphenyl, + bibenzyl, + ethylbenzene, + 1,4-dimethylbenzene, and + benzoic acid; anthracene + *o*-terphenyl, + *m*-terphenyl, + *p*-terphenyl, and + bibenzyl; and biphenyl + chrysene, + pyrene, + fluoranthene, + toluene, + ethylbenzene, + 1,4-dimethylbenzene, + butanone, + phenol, + benzoic acid, and + ethyl acetate. For these systems, it is possible to predict the SLE curves with SAFT-γ Mie (e.g., assuming the mixtures to be eutectic systems) but no AAD can be included in the heatmap.

The mixture of anthracene + fluorene has been either described as a eutectic system^120^ or as completely miscible in the solid phase.^15^ The same inconsistency is found for anthracene + acenaphthene, described as eutectic^120^ or completely miscible^317^ according to different studies. For both mixtures, we represent the results on the heatmap for the eutectic description. We give the AADs for both descriptions in Table 5 as well as the quantitative information about the accuracy of the SLE calculations obtained for all the systems considered. The overall accuracy for other properties (vapor pressure, density of pure fluids, vaporization enthalpy, bubble and dew temperature and pressure, density of binary mixtures, and excess enthalpy) is summarized in Table 6. Finally, an overview of the accuracy per property is provided in Table 7.

**Table 7:** Overview of the Accuracy of SAFT-*γ* Mie Calculations for the Pure Fluids and Binary Mixtures Considered in Our Work^a^

property	*N*_*p*_^tot^	%AAD	AAD
decimal logarithm of vapor pressure	392	2.285	0.07777
liquid density (pure fluids)	173	2.954	24.77 kg/m^3^
enthalpy of vaporization	130	6.334	3.292 kJ/mol
bubble temperature	563	0.7643	2.960 K
dew temperature	342	0.3366	1.157 K
decimal logarithm of bubble pressure	574	0.7919	0.03519
decimal logarithm of dew pressure	246	0.7228	0.04145
liquid density (mixtures)	697	1.004	8.664 kg/m^3^
excess molar enthalpy	271	18.14	96.86 J/mol
SLE composition	2588	N/A	0.05515

a *N*_*p*_^tot^ is the number of experimental data for each property *p*.

## 4 Conclusions

The thermodynamic properties and phase behavior of polycyclic aromatic hydrocarbons (PAHs) in numerous organic solvents have been modeled with the SAFT-γ Mie group-contribution approach. We have extended the applicability of SAFT-γ Mie by developing 33 new unlike group interactions. The accuracy of the calculations has been evaluated by comparing them to experimental data through phase diagrams across a wide range of thermodynamic conditions, as well as by calculating the corresponding %AADs and AADs. We have obtained excellent agreement between the SAFT-γ Mie calculations and the experimental data for all of the mixtures and the thermodynamic properties considered.

Several notable features in the thermodynamic properties have been observed by comparing the available experimental data with the SAFT-γ Mie calculations. One concerns the binary mixtures including chrysene. The mixture of chrysene + pyrene forms a eutectic system, while the mixture of chrysene + benz­[*a*]­anthracene forms a single solid phase. Both chrysene and benz­[*a*]­anthracene have been modeled with identical SAFT-γ Mie groups, differing only in their melting properties. The model leads to an ideal solid–liquid equilibrium (SLE) for chrysene + benz­[*a*]­anthracene, which closely matches experimental data. The new unlike interactions involving the functional groups found in aromatic hydrocarbons (aC, aCH, aCCH_3_, and aCCH_2_) and unsaturated aliphatic compounds (C=, CH=, and CH_2_=) have been integrated into the existing database of SAFT-γ Mie parameters. This enables future prediction of the thermodynamic properties and phase behavior of compounds and mixtures that are relevant for many industries (oil, energy, food, pharmaceutic, fragrance, etc.) and environmental concerns (e.g., air and water pollution), considering a broad range of temperatures, pressures, and compositions. In a pharmaceutical context, the new groups are relevant for modeling compounds such as aspirin and paracetamol (that can be modeled with the aC group) and can guide the development of new active pharmaceutical ingredients, formulations, and processes.

## Data Availability

Data underlying this article can be accessed on Zenodo at https://zenodo.org/records/21792020 and used under the Creative Commons Attribution license.
